# Hydrogen Peroxide Fuel Cells and Self-Powered Electrochemical Sensors Based on the Principle of a Fuel Cell with Biomimetic and Nanozyme Catalysts

**DOI:** 10.3390/bios15020124

**Published:** 2025-02-19

**Authors:** Yunong Zhang, Yuxin Liu, Andreas Offenhäusser, Yulia Mourzina

**Affiliations:** 1 Institute of Biological Information Processing—Bioelectronics (IBI-3), Forschungszentrum Julich, 52425 Julich, Germany; yun.zhang@fz-juelich.de (Y.Z.); y.liu@fz-juelich.de (Y.L.); a.offenhaeusser@fz-juelich.de (A.O.); 2 Faculty of Mathematics, Computer Science and Natural Sciences, RWTH Aachen University, 52062 Aachen, Germany

**Keywords:** hydrogen peroxide, electrochemical sensor, biosensor, fuel cell, self-powered electrochemical sensors, nanozyme, biomimetic material, Prussian blue, phthalocyanine

## Abstract

The operating principle of a fuel cell is attracting increasing attention in the development of self-powered electrochemical sensors (SPESs). In this type of sensor, the chemical energy of the analyzed substance is converted into electrical energy in a galvanic cell through spontaneous electrochemical reactions, directly generating an analytical signal. Unlike conventional (amperometric, voltammetric, and impedimetric) sensors, no external energy in the form of an applied potential is required for the redox detection reactions to occur. SPESs therefore have several important advantages over conventional electrochemical sensors. They do not require a power supply and modulation system, which saves energy and costs. The devices also offer greater simplicity and are therefore more compatible for applications in wearable sensor devices as well as in vivo and in situ use. Due to the dual redox properties of hydrogen peroxide, it is possible to develop membraneless fuel cells and fuel-cell-based hydrogen peroxide SPESs, in which hydrogen peroxide in the analyzed sample is used as the only source of energy, as both an oxidant and a reductant (fuel). This also suppresses the dependence of the devices on the availability of oxygen. Electrode catalyst materials for different hydrogen peroxide reaction pathways at the cathode and the anode in a one-compartment cell are a key technology for the implementation and characteristics of hydrogen peroxide SPESs. This article provides an overview of the operating principle and designs of H_2_O_2_–H_2_O_2_ fuel cells and H_2_O_2_ fuel-cell-based SPESs, focusing on biomimetic and nanozyme catalysts, and highlights recent innovations and prospects of hydrogen-peroxide-based SPESs for (bio)electrochemical analysis.

## 1. Introduction

The experimental work of W. R. Grove on the “gas voltaic battery” was later conceptualized by F. W. Ostwald in his idea of chemical-to-electrochemical energy transformation [1] and was subsequently realized in various kinds of future electrochemical fuel cells (FCs) [2]. In 2001, the concept of generating electrical energy using (bio)electrochemical reactions taking place in biofuel cells (BFCs) was realized in a new type of glucose and lactate electrochemical biosensor–self-powered bioelectrochemical sensor (SPBioES) [3] based on a design of a non-compartmentalized glucose—O_2_ biofuel cell and bioengineered electrode surfaces [4]. These sensors illustrated the concept of developing electrochemical sensors based on the fuel cell principle. The first SPBioES demonstrated the dependence of the open-circuit voltage on the concentration of glucose or lactate analyte [3]. The SPBioESs based on generating and recording current changes have since become more widespread, although registration of the open-circuit potential has the advantage of measuring at conditions when no current is passed through the sample. It might be more acceptable for biological or other sensitive samples, for example [5,6,7].

Traditional electrochemical sensors use voltammetric, amperometric, or impedimetric techniques operating in a three-electrode electrochemical cell under the control of an external power supply to apply and control the potential on the working electrode for a particular redox indicator reaction to occur. This configuration makes flexible designs and practical applications difficult. To avoid the use of an external power system and to minimize the number of electrochemical cell components involved, fuel-cell-based SPESs based on a combination of selected thermodynamically favorable reactions are attracting increasing attention. Operating in a two-electrode configuration, SPESs generate open-circuit potential or short-circuit current depending on the concentration of analyte avoiding the power supply and modulation system. For the detection of various analytes, SPBioESs and SPESs have therefore become particularly interesting and relevant types of sensors in the field of (bio)electrochemical analysis. Extensive research has focused on translating the advantageous fuel cell principle to wearable and implantable power supply and sensor systems, and there are a number of reviews on these topics [7,8,9,10,11,12,13,14,15,16,17,18].

Although the operating principle of the fuel cell and of the corresponding SPES on which it is based are similar, high electrical power densities—which are desirable for the fuel cell to be applied as a source of energy—are not necessarily a requirement for the SPES. On the one hand, low current and electrical power densities in SPESs may eliminate the redox transformations of interfering substances and reduce damage to the sample. Nevertheless, high current and OCP values might be favorable for achieving high sensitivities for the SPES.

One particularly interesting application of electrochemical sensors is the electrochemical analysis of hydrogen peroxide. Hydrogen peroxide plays an important role in various industrial, environmental, and physiological processes. It has achieved widespread commercial use as an oxidant, disinfectant, or bleaching agent. It is worth noting that H_2_O_2_ is naturally present in humans and plants as one of the reactive oxygen species (ROS) and a product of metabolism as well as being produced in aquatic systems due to sunlight. The term ROS is most often expanded to include both highly reactive free oxygen radicals (e.g., superoxide anion (O_2_^●−^) and the hydroxyl radical (●OH)) and non-radical ROS such as more stable “diffusable” hydrogen peroxide and singlet oxygen (^1^O_2_), while hydrogen peroxide is capable of producing radical ROS such as superoxide and hydroxyl radicals [19,20,21]. Hydrogen peroxide electrochemical sensors have therefore been developed for various applications, including biomedical diagnostics and health monitoring, industrial processes, food safety, agriculture, and environmental monitoring [22,23,24,25,26,27]. Moreover, hydrogen peroxide sensors are used in combination with biological recognition elements such as enzymes in bioelectrochemical sensors, where hydrogen peroxide is a product of oxidoreductase-catalyzed reactions and its quantification is used to detect important biomarkers such as glutamate, lactate, uric acid, and glucose. Along with its significance in analytical chemistry, hydrogen peroxide has also been considered a carbon-free clean energy carrier in hydrogen peroxide FCs (HPFCs) and as an oxidant in various types of FCs and BFCs [28,29,30]. Although progress has been achieved in the development of electrochemical H_2_O_2_ sensors based on various electrocatalysts, research into hydrogen peroxide SPESs is still at an early stage.

Dual redox properties of hydrogen peroxide make it possible to develop membraneless, one-compartment, fuel-cell-based H_2_O_2_ SPESs using H_2_O_2_ as both a reductant and an oxidant. To achieve this goal, pioneering work has been undertaken with different electrode catalyst materials such as noble metals and enzymes as well as biomimetic materials and nanozymes. It is worth noting that the application of natural enzymes and bioelectrodes can increase costs, limit operation and storage conditions, and harm long-term stability, and often requires mediated electron transfer or molecular engineering to improve the electron transfer in bioelectrocatalysis [13,31], while the use of noble metal catalysts is expensive and often not selective. It is therefore necessary to study non-enzymatic materials such as biomimetic materials and nanozyme catalysts to avoid the disadvantages, while also maintaining good catalytic activity and selectivity.

Biomimetic materials imitate or mimic the functions, mechanisms, or models of biomolecules and biological systems. Among them, in particular, are “artificial enzymes” or “enzyme mimics”, which are synthetic catalysts “imitating catalytic processes that occur in living systems” [32,33], i.e., substances with enzyme-like catalytic activity. Later, as a result of the development of nanoscience and functional nanomaterials, the term nanozyme appeared to describe nanomaterials with enzyme-like catalytic activity [34]. This term became popular after the publication of a review by Wei and Wang [35], in which nanozymes were defined as nanomaterials with enzyme-like activity, thus placing nanozymes in the context of “artificial enzymes” [36]. Biomimetic materials and nanozymes have attracted considerable attention due to the expectations of synergy between enzyme functionalities, better stability, broad application conditions, and a realization of a direct electron transfer. Various types of biomimetic materials and nanozymes have been investigated as mimics of horseradish peroxidase (HRP) in the reduction of hydrogen peroxide, such as metal and carbonaceous nanomaterials, metal oxides, transition metal complexes, and metal–organic frameworks. The types, methods of synthesis, applications, and progress of biomimetic materials and nanozymes can be found in numerous reviews on this topic [33,35,37,38,39].

Moreover, since the concentration of H_2_O_2_ is not high in many analytical applications (usually in the nM to mM range), the activity of electrode materials of the H_2_O_2_ SPESs plays a key role in sensor functionality. It is therefore important to improve the sensor properties, especially the current density, to obtain high sensitivity. Thus, the development of new electrode catalyst materials with improved characteristics is one of the key ways of improving the efficiency of chemical power sources based on fuel cells and the characteristics of sensors based on them. Based on the reasons above, in recent years, studies have been performed on biomimetic and nanozyme catalysts.

In this article, we first review the development of peroxide–peroxide fuel cells (HPFCs), where H_2_O_2_ is used as both oxidant and reductant (or “fuel”), which became the starting point for the development of H_2_O_2_ SPESs. The electrode catalysts are also discussed, since the properties of hydrogen peroxide SPESs are primarily determined by the catalytic materials that impart specificity and catalytic activity to the sensor systems. However, hydrogen peroxide fuel cells, where hydrogen peroxide is only used as an oxidant [28,40], are not included, with the exception of a number of particular examples. Some closely related aspects that expand the application of HPFCs, such as sustainable hydrogen peroxide fuel cells, SPPhotoESs, single-enzyme SPBioESs, the replacement of the oxygen cathode, and SPESs with optical readout, are also considered.

## 2. Mechanism of HPFCs

**Figure 1:**
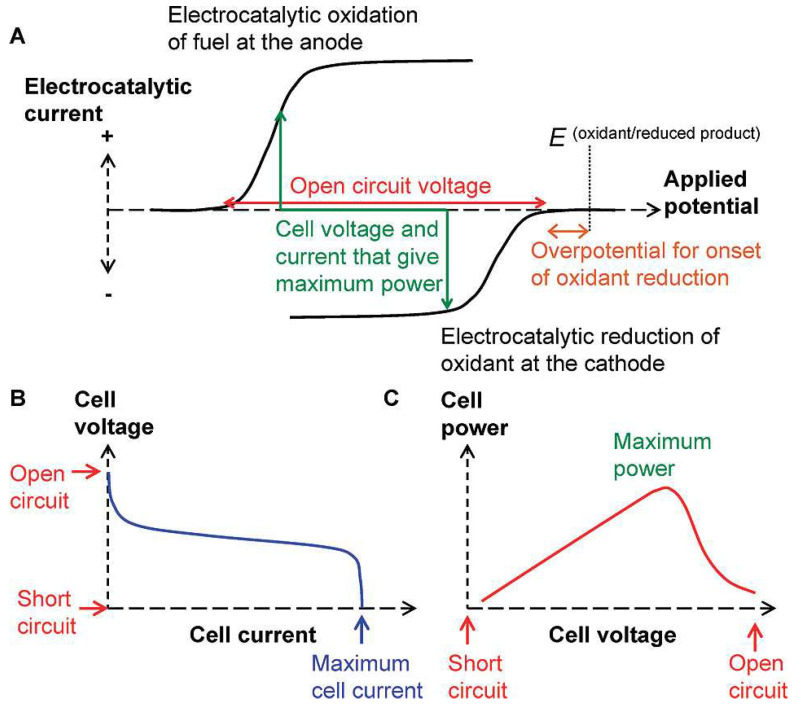
(**A**) Characteristics of the cathode and anode for the construction of an FC; (**B**,**C**) parameters that determine the efficiency of an FC. Reprinted with permission from [43]. Copyright 2008 American Chemical Society.

Figure 1 illustrates the working principle of the FC and SPES based on a galvanic cell. The cell voltage during current flow (discharge potential) can be expressed as Equation (1) [2,41]:

E_d_ = E_0_ − η_a_ − |η_c_| − I⋅ΣR = E_c_ − E_a_ − η_a_ − |η_c_| − I⋅ΣR > 0, (1)

where E_d_ is the cell voltage with current flow (discharge potential); E_0_ = E_c_ − E_a_ is the cell voltage in the absence of current flow in the cell, representing open-circuit voltage (OCV); E_c_ and E_a_ are the cathode and anode potentials; η_c_ and η_a_ represent the polarization of the cathode and anode; I is the current in the operating mode of the cell; and ΣR is the sum of internal and external resistances, except for polarization resistances taken into account by η. The criterion for the operation of a fuel cell with spontaneously occurring thermodynamically favorable reactions is the negative change of Gibbs energy and, consequently, E_d_ > 0 [2,42]. Accordingly, the suitability of cathode and anode reactions for building a fuel cell can be initially assessed using “onset potentials” derived from the I–E curves of the reductant and oxidant, as illustrated in Figure 1A [43,44], using Equation (2):

E_OCV_ = E_c_^onset^ − E_a_^onset^ > 0 (2)

**Figure 2:**
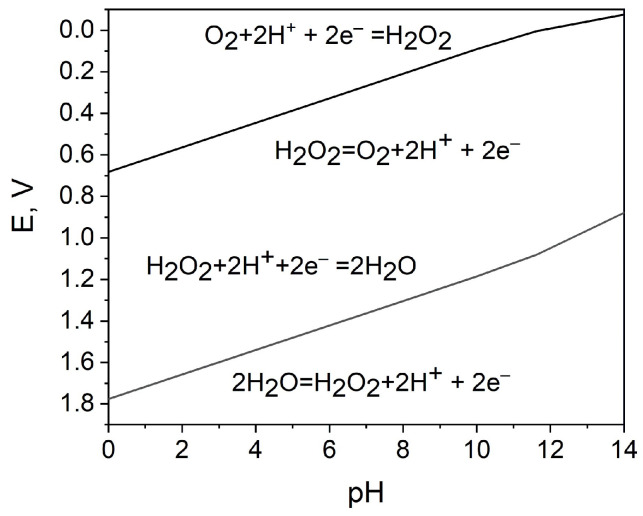
Potential−pH diagram for the characterization of the oxidizing and reducing action of hydrogen peroxide; the data for 1 M hydrogen peroxide are calculated using [45].

As depicted in Figure 1 and Equations (1) and (2), for the development of an H_2_O_2_ SPES, it is essential that the reduction potential of H_2_O_2_ on the cathode be more positive than the oxidation potential of H_2_O_2_ on the anode. To this point, Pourbaix diagrams [45] are applicable references, as they describe the dependence of the redox properties of hydrogen peroxide on its concentration and pH (Figure 2).

Accordingly, the hydrogen peroxide reduction and oxidation reactions in the acidic medium are as follows:

Reduction: H_2_O_2_ + 2H^+^ + 2e^−^ → 2H_2_O, at pH 0 : E^0^_c_ = 1.78 V vs. SHE (3)

Oxidation: H_2_O_2_→ O_2_ + 2H^+^ + 2e^−^, at pH 0 : E^0^_a_ = 0.68 V vs. SHE, (4)

where E^0^_c_ and E^0^_a_ are the standard electrode potential of the hydrogen peroxide reduction and oxidation reactions, respectively [45].

The hydrogen peroxide reduction and oxidation reactions in neutral or alkaline electrolytes are [46,47]:

Reduction: HO_2_^−^ + H_2_O + 2e^−^ → 3OH^−^, at pH 14: E^0^_c_ = 0.88 V vs. SHE (5)

Oxidation: HO_2_^−^ + OH^−^ → O_2_ + H_2_O + 2e^−^, at pH 14: E^0^_a_ = –0.075 V vs. SHE (6)

The overall reaction can be expressed as Equation (7), although the mechanisms in acidic and alkaline media are different and the oxidant properties of H_2_O_2_ are more pronounced in acidic medium:

2H_2_O_2_ → 2H_2_O + O_2_ (7)

A potential difference between H_2_O_2_ reduction reaction and oxidation reaction provides a theoretical background for HPFCs, where H_2_O_2_ can be used separately as the oxidant and the reductant [28]. E^0^_c_ − E^0^_a_ > 0 can thus be established due to this potential difference. Since it is thermodynamically advantageous to use hydrogen peroxide as an oxidant in an acidic electrolyte (reaction (3)), and hydrogen peroxide as a reductant in an alkaline electrolyte (reaction (6)) to build HPFCs and SPESs, they can be realized in a two-compartment, membrane-separated cell design. However, for sensor applications, a one-compartment membraneless system is more attractive because of the simplified structure and convenient operation. By selecting appropriate catalysts, the cell configuration can be simplified to a one-compartment cell without a membrane. Accordingly, selective electrode catalysts are key elements for the development of hydrogen peroxide FCs and SPESs.

## 3. Development of HPFCs

Hydrogen peroxide has been discussed as an ideal energy carrier alternative to hydrocarbon energy sources and hydrogen, as it can be used in HPFCs to produce electricity and reduce the dependence on fossil fuels. It is also a clean energy source that does not emit greenhouse gases [29,48,49,50]. Compared to conventional H_2_/O_2_ fuel cells, HPFCs have higher safety due to the aqueous storage and transportation of hydrogen peroxide instead of gaseous hydrogen. Nevertheless, it should be noted that at present, the power density of H_2_/O_2_ fuel cells exceeds more than 1 W cm^−2^, but HPFCs have a much lower power density (~mW cm^−2^), which makes HPFCs not suitable for large-scale energy devices. Meanwhile, the stability of hydrogen peroxide should be taken into consideration. To avoid decomposition, hydrogen peroxide should not be exposed to metals and sunlight. HPFCs can theoretically achieve an output potential of 1.09 V in acidic electrolytes, which is close to that of the methanol/air (1.21 V) and H_2_/air (1.2 V) FC. HPFCs also offer the advantages of using H_2_O_2_ as both an oxidant and a fuel in a simple one-compartment membraneless cell as well as easier storage of the fuel/oxidant. In addition, HPFCs can work in an air-free or oxygen-deficient environment such as under water or in outer space, and can generate carbon-free electricity while producing water and oxygen and being environmentally friendly. Moreover, hydrogen peroxide can be produced using sustainable energy sources, which means that HPFCs support energy sustainability. HPFCs have therefore been extensively studied. The properties, advantages, and perspectives of H_2_O_2_ as a clean energy fuel were reviewed in [28,29,49,51].

### 3.1. Two-Compartment Design of HPFCs

**Figure 3:**
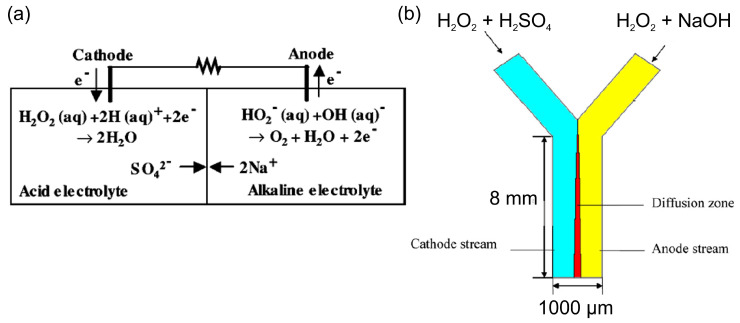
(**a**) Scheme and operating principle of the two-compartment HPFC [46]. © The Electrochemical Society. Reproduced with permission from IOP Publishing Ltd.(Bristol, UK). All rights reserved. (**b**) Scheme of a membraneless Y-shaped microfluidic HPFC, where hydrogen peroxide is reduced and oxidized in different electrolytes of the cathode and anode streams, respectively. Reprinted from [59] with permission from Elsevier (Amsterdam, The Netherlands).

Table 1 summarizes the development of HPFCs and HPFC-based SPESs in chronological order. Following Section 2, the first HPFC fuel cells based on Pt and Pd catalysts and a two-compartment design with acidic catholyte and alkaline anolyte showed relatively good performance in terms of open-circuit potential (OCP) and maximum power density (MPD) [46,52,53,54,55,56,57] (Table 1). The first cell for electricity generation based on the electrochemical decomposition of hydrogen peroxide included Pt electrodes and achieved a high OCP value of 0.7 V, an MPD of 23 mW cm^−2^, and a maximum current density (MCD) of about 80 mA cm^−2^ due to the high electrocatalytic activity of this electrode material and the acidic–alkaline electrolyte compartments (Figure 3a) [46]. Later on, Yang et al. [54] used a pulsed electrodeposition technique to produce nanodendritic metal structures and nanowires [24] for the preparation of the high-surface-area nanodendritic Pd catalysts. HPFCs with dendritic Pd electrodes showed a high OCP of 0.9 V and an MPD of 14.3 mW cm^−2^ at 20 °C [54]. As can be seen in Table 1, along with different pH values, a higher concentration of H_2_O_2_ was often used in catholytes to achieve higher oxidizing action of hydrogen peroxide [52,54,55,56,57,58]. Through modification of the catalysts and electrode materials, better performance of the HPFC was obtained by the same group. It was reported that HPFCs with a Pd/carbon fiber cloth (CFC) cathode in combination with an Ni/CFC anode showed an OCP of 0.9 V and an MPD of 21.6 mW cm^−2^ at 20 °C [55]. They also used an Au–Pd nanocomposite on CFC as electrodes [58] in a similar FC with an OCP of 0.9 V and an MPD of 20.7 mW cm^−2^ at 20 °C. Significantly higher MPD was achieved by the same group by using a 3D Ni anode and a Pd/CFC cathode; the HPFC showed an OCP of 0.9 V and an MPD of 48.7 mW cm^−2^ at 20 °C [56]. It is worth noting that due to the difference in the catholyte and the anolyte in this two-compartment design of an HPFC, a membrane is necessary to separate the two electrolyte solutions.

Attempts to eliminate the use of a membrane were made with a membraneless micro-fuel cell design, which utilized the nature of laminar flow in a Y-shaped microchannel [59]. In this cell, the cathode and anode electrolytes flowed in parallel through the microchannel without turbulent mixing. On the one hand, this cell had the advantage of a two-compartment cell, which allowed for the reduction of hydrogen peroxide in an acidic catholyte and the oxidation of hydrogen peroxide in an alkaline anolyte. On the other hand, this design allowed for the elimination of the use of a membrane due to the partition of two electrolytes flowing at low Reynolds numbers (Figure 3b). The authors modeled the performance of such a microfluidic cell and found it was in good agreement with the experimental data obtained in a two-compartment membrane cell by Hasegawa [46].

The electrochemical reactions of H_2_O_2_ decomposition on the cathode and anode not only make the HPFC a clean electricity generator, but also make it possible to develop an H_2_O_2_ SPES. Nevertheless, as expected from thermodynamic considerations and confirmed by the experimental results in Table 1, it should be noted that the best performance of the two-compartment HPFCs was generally achieved when using an acidic catholyte, an alkaline anolyte, and high concentrations of H_2_O_2_, which is not the case for SPESs since the analyte concentrations are relatively low and the test medium is limited by sample conditions. For SPESs, a one-compartment configuration and milder conditions are most relevant. Moreover, the use of electrocatalysts other than precious metals would make SPESs cost-effective.

Since electrocatalysts are a key technology for the performance and characteristics of fuel cells and sensors, enabling specific tasks to be solved selectively, these shortcomings have been further addressed by finding new inexpensive electrocatalysts. These electrocatalysts ensure that selectivity and good performance are achieved not only by adjusting the pH but also by selective oxidation and reduction of hydrogen peroxide at relatively low concentrations in one solution and under mild conditions.

### 3.2. One-Compartment HPFC and the Use of Biomimetic and Nanozyme Catalysts

**Figure 4:**
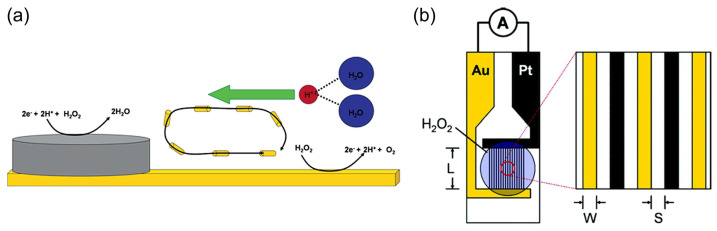
(**a**) Scheme of a bipolar particle with an Ag cathode area for hydrogen peroxide reduction and an Au anode area for hydrogen peroxide oxidation. Reprinted with permission from [60]. Copyright 2005 American Chemical Society. (**b**) A scheme of a Pt/Au IDME for the measurement of the current between the Pt anode and Au cathode due to the electrocatalytic decomposition of hydrogen peroxide. Reprinted with permission from [61]. Copyright 2006 American Chemical Society.

Sen et al. [60,61] used selective electrocatalytic decomposition of hydrogen peroxide to water and oxygen based on the principle of galvanic electrochemistry in one “cell” on bipolar metal nanoparticles (for example, Pt–Au or Ag–Au) to generate a proton gradient, flow in microfluidic systems, and motion of particles (Figure 4a). The authors also found [61] the dependence of the steady-state current density of the short-circuited Pt and Au interdigitated microelectrodes (IDMEs) on the concentration of hydrogen peroxide (Figure 4b), although the fuel cell application was not demonstrated in these works.

When using metal electrodes with a lower electrocatalytic activity towards the hydrogen peroxide redox reactions in a one-compartment configuration, the OCP and the performance of the first HPFCs were relatively low [62,63] compared to the two-compartment cells with Pt and Pd catalysts, as described in Section 3.1. Yamazaki et al. [62] reported on a membraneless, one-compartment HPFC with an Au plate as an anode and an Ag plate as a cathode in an alkaline electrolyte. The OCP of the cell was only about 0.095 V. A similar one-compartment HPFC was further combined with the production of hydrogen peroxide using sunlight [63]. Instead of using an Ag plate as a cathode, Ag-Pb alloy nanoparticles (NPs) were used for the construction of a one-compartment HPFC, since a higher specific surface area of the Ag catalyst was expected for the Ag NPs compared to an Ag plate. Although the performance was slightly improved, the OCP was only about 0.15 V and the MPD was 75 µW cm^−2^.

Yamada et al. (2011) [64], Wong et al. (2011) [65], and Shaegh et al. (2012) [66] began investigating transition metal complexes as biomimetic materials and nanozyme catalysts in HPFCs. Wong et al. [65] also reported on the first demonstration of an H_2_O_2_ SPES based on an HPFC, although the FC had two compartments and only used H_2_O_2_ as an oxidant, while the anode reaction was the oxidation of water producing oxygen. In most studies exploring these biomimetic catalysts and nanozymes, acidic electrolytes were used because of the improved catalytic activity of the catalyst [64,67] and the stability of Prussian blue (PB) [68,69,70] as well as the improved stability of H_2_O_2_ due to a lower ionization rate to HO_2_^−^ [28,71].

Yamada et al. [64] reported on a one-compartment fuel cell with an Ni anode and a protonated iron phthalocyanine complex as a cathode catalyst working under mild acidic conditions (pH 3). The OCP of the cell at relatively milder conditions than in previous works was 0.5 V and the MPD was 10 µW cm^−2^ at 0.3 M H_2_O_2_. A Nafion coating on both the cathode and the anode was used to suppress the leaching of the catalysts and to improve cell performance. Shaegh et al. [66] exploited the “artificial peroxidase” PB-modified cathode for the one-compartment HPFC. In combination with an Ni anode, an OCP of 0.6 V and an MPD of 1.55 mW cm^−2^ were achieved at 0.5 M H_2_O_2_. However, this higher MPD value was achieved in a more acidic solution (0.1 M HCl) using a slightly higher concentration of H_2_O_2_ (0.5 M) than in the previous work (0.3 M H_2_O_2_) on a protonated iron phthalocyanine complex as a cathode catalyst.

Most H_2_O_2_ fuel cells reported so far have since included a cathode based on two types of transition metal complexes (TMCs): cyanobridged assemblies, such as PB and its analogues, and metallophthalocyanines (MPcs), since these substances possess high catalytic activities for the hydrogen peroxide reduction reaction. For example, Prussian blue and PB analogues have been referred to as “artificial peroxidase” and TMC nanozymes due to their ability to effectively substitute peroxidase enzyme in a hydrogen peroxide reduction. MN4 complexes (metalloporphyrins and metallophthalocyanines) are traditionally considered biomimetic compounds due to the similarity of their structure and some redox and catalytic properties to heme proteins such as cytochrome *c*, horseradish peroxidase, and hemoglobin. The mechanism of these electrocatalysts has been extensively studied [67,69,72,73]. To achieve better performance, carbonaceous nanomaterials have often been used for the modification of electrodes, with the aim of increasing the electroactive area of the electrodes and improving the dispersion of the catalysts, which allows for an increase in the number of electrocatalytic sites. Moreover, carbonaceous nanomaterials improve the conductivity of the interface and the electron transfer between the catalyst and the electrode surface. Therefore, modifications of MPc- and PB-based TMC biomimetic materials and nanozymes with carbonaceous nanomaterials have been widely studied and employed in electrochemical processes and devices. Other than cathode catalyst materials, among all the anode materials, Ni and Ag have been the mostly widely used anode materials for H_2_O_2_ fuel cells and SPESs so far. Table 1 presents configurations and properties of the HPFC and recent H_2_O_2_ SPES. However, the choice and mechanism of the anode material has not received proper attention so far.

**Figure 5:**
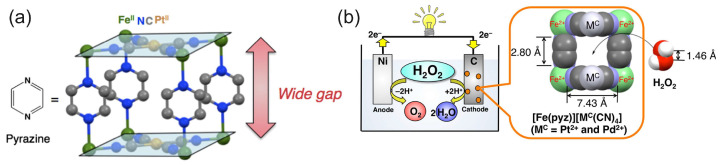
(**a**) Structure of the pyrazine-bridged Fe[Pt(CN)_6_] with a wide gap accessible for hydrogen peroxide. Reprinted from [48] with permission from Elsevier. (**b**) The pyrazine-bridged Fe[Pt(CN)_6_] as a cathode material for the HPFC. Reprinted with permission from [75]. Copyright 2014 American Chemical Society.

The study by Yamada et al. [74] further investigated the PB analogues as cathode electrocatalysts for the HPFC using a series of polynuclear cyanide complexes of the first-row transition metals Fe, Co, Mn, and Cr. In a simple cubic lattice of PB, alternating Fe(II) and Fe(III) are linked by linear cyanide groups, with carbon atoms coordinated to the Fe(II) [68]. Based on the comparative results of the fuel cell performances, the authors identified the N-bound Fe ions as the active species in the H_2_O_2_ reduction and concluded that stable Fe^2+^–N bonds were crucial to the high performance of the Fe^II^_3_[Co^III^(CN)_6_]_2_ catalyst and HPFC. A cell comprising Fe^II^_3_[Co^III^(CN)_6_]_2_ on carbon cloth as a cathode and Ni as an anode achieved a high OCP of 0.68 V and an MPD of 0.45 mW cm^−2^ in pH 3 with 0.3 M H_2_O_2_ and 0.1 M NaCl. When operating at pH 1 (HClO_4_, 0.3 M H_2_O_2_, and 0.1 M NaCl), the OCP was 0.78 V and the MPD was about 1 mW cm^−2^. Based on the previous finding on the importance of the nitrogen-coordinated Fe^2+^ ions as an active species in the reduction of H_2_O_2_, the same authors further increased power density by increasing the number of Fe^2+^ ions available for interaction with H_2_O_2_, using pyrazine as an additional ligand [75]. This ligand was used to increase the spatial accessibility of Fe^2+^ ions for H_2_O_2_ molecules (Figure 5). Moreover, since pyrazine is a weakly bound ligand, the Fe^2+^ ions were expected to be able to interact with H_2_O_2_. At pH 1, the OCP of the cell with the porous pyrazine-bridged Fe[Pt(CN)_4_] catalyst was about 0.7 V and the power density reached 4.2 mW cm^−2^, which was the highest value for the one-compartment HPFC reported at that time. Similar results were obtained for the pyrazine-bridged Fe[Pd(CN)_4_]. Low acidity of the electrolyte resulted in a lower power density of about 0.09 mW cm^−2^ (as shown for the pyrazine-bridged Fe[Pt(CN)_4_] catalyst). Investigations of a series of cyanometallate catalysts thus demonstrated that the performance of the HPFC was dependent on the coordination sphere of Fe^2+^ ions [48]. The performance of the HPFC with a [Fe^II^(H_2_O)_2_]_3_[Co^(III)^(CN)_6_]_2_ cathode and an Ni anode was further improved by the addition of Sc^3+^ ions to the electrolyte of the HPFC [76]. It was assumed that Sc^3+^ suppressed the decomposition of H_2_O_2_ and lowered the pH. It was also found that heat treatment of [Fe^II^(H_2_O)_2_]_3_[Co^(III)^(CN)_6_]_2_ at 120 °C resulted in the unfavorable oxidation of Fe^II^. The cell had an OCP of 0.81 V and an MPD of 9.9 mW cm^−2^. In addition to the OCP and MPD, the durability of the cell was also improved in the presence of Sc^3+^ ions. When no Sc^3+^ ions were present in the electrolyte, the output potential dropped from 0.3 V (operation at the constant current of 2 mA cm^−2^) to 0 V within 5 min, while in the presence of 0.1 M Sc^3+^ ions, the potential only decreased by 0.04 V after 90 min of operation.

Pariyar et al. [77] designed a phenalenyl-based Fe(III) complex with electrocatalytic hydrogen peroxide reduction properties and used it as a cathode material for a one-compartment HPFC. Miglbauer et al. [78] compared PEDOT:PSS and PEDOT:PSS+PB as cathode materials for the HPFC in combination with an Ni anode in acidic media. It was shown that PEDOT:PSS can be used as a cathode material on its own. The addition of PB resulted in a slight increase in the OCP from 0.58 V to 0.64 V, but a higher power density was achieved for the PEDOT:PSS cathode without PB.

**Figure 6:**
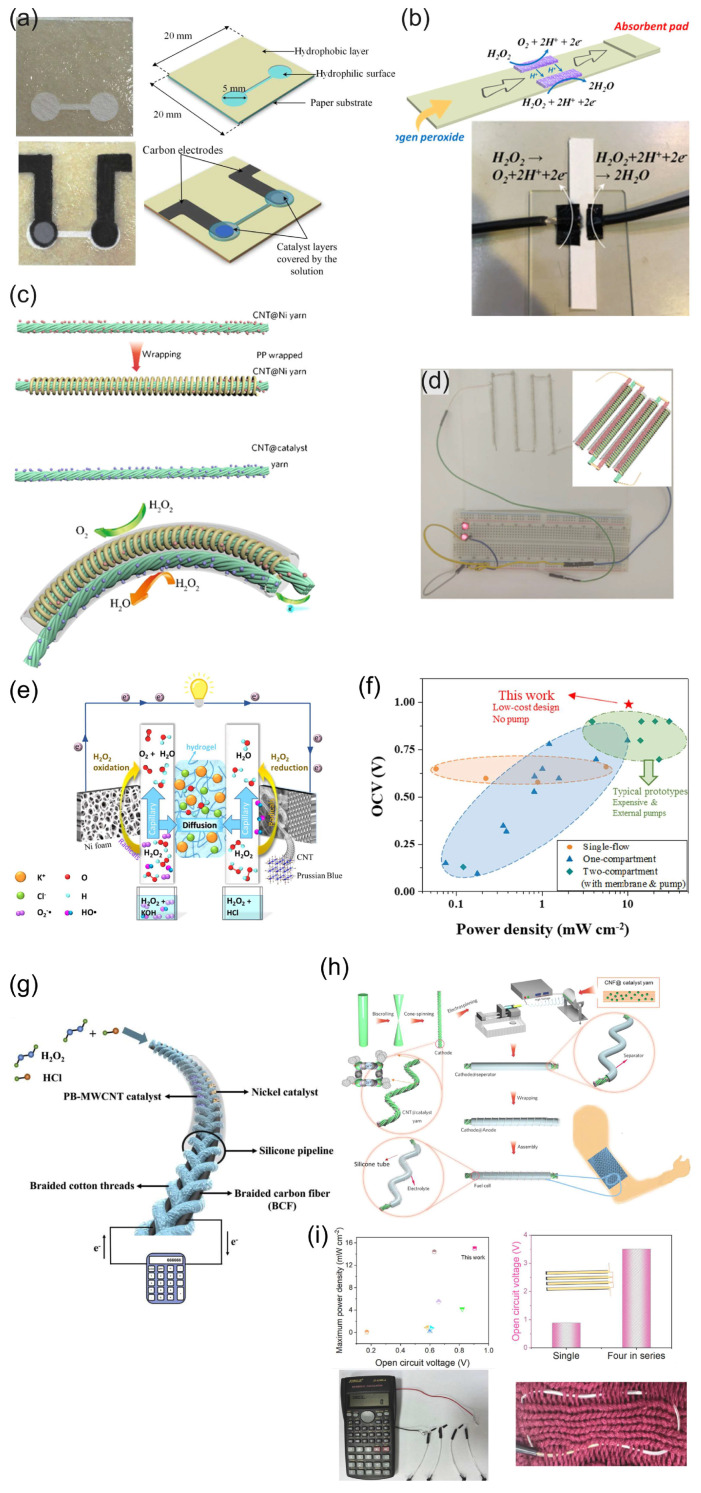
(**a**) Images of the paper-based H_2_O_2_ membraneless µ-fuel cell showing the patterned hydrophilic and hydrophobic surfaces, electrodes, and catalysts. Reprinted from [80] with permission from Elsevier. (**b**) Schematic and photograph of a paper-based µ-fluidic HPFC. Reproduced from [81] with permission from WILEY. (**c**) Schematic of the flexible yarn-based cathode, anode, and _W_HPFC, and (**d**) LED powered by four _W_HPFCs connected in series. (**c**,**d**) Reproduced from [83] with permission from WILEY. (**e**) Illustration of a paper-based pumpless µ-fluidic HPFC with a “gel-aided” two-electrolyte configuration, and (**f**) comparison of the parameters of the “gel-aided” HPFC with the parameters of the HPFC reported so far. (**e**,**f**) Reprinted from [87] with permission from Elsevier. (**g**) Schematic of the flexible membrane-less µ-fluidic “on-fiber” HPFC. Reprinted from [85] with permission from Elsevier. (**h**) Schematic illustration of the fabrication of the coaxial fiber HPFC, and (**i**) properties of the coaxial fiber HPFC and example of the use of a coaxial fiber HPFC cell as a power source for a calculator. (**h**,**i**) Reprinted from [86] with permission from Elsevier.

Since the cathode catalytic materials of HPFC are generally supported by conductive materials, the variety of conductive substrates brings possibility for the development of flexible HPFCs, as flexible and wearable fuel cells have received enormous attention as candidates for wearable energy devices and the next generation of wearable electronics. Flexible fuel cells can be built on various layered structures, and there is also great potential and interest in wearable electronic and sensor devices that are built on fibers and fiber-based clothing [79]. Consequently, PB and its analogues (with and without carbonaceous nanomaterials) were further used as cathode materials in combination with Ni or Ag anodes mostly in strongly acidic media to prepare the FC in various flexible designs—for example, paper, flexible microfluidics, or wire-shaped flexible fuel cells (“_W_FCs”) [80,81,82,83,84,85,86] (Figure 6 and Table 1). A paper-based H_2_O_2_ micro-FC with the PB cathode catalyst was used in [80] (Figure 6a). In this work, the PB and Al or Ni slurry (anode) were deposited onto carbon electrodes, which were screen printed on the paper. PB and Al or Ni were used as mixtures with Nafion. The filter paper was patterned by SU-8 to form hydrophilic and hydrophobic surfaces (microfluidic channel with open electrode surfaces and connection channel). The OCP of the cells operated in 0.1 M HCl with 0.5 M H_2_O_2_ was 0.32 V and 0.61 V, and the MPD was 0.38 mW cm^−2^ and 0.81 mW cm^−2^ with Ni and Al anodes, respectively. An increase in the H_2_O_2_ concentration to 1 M resulted in a roughly 20% increase in the MPD. It was found that the degradation of the cell response during operation resulted from the formation of oxygen bubble on the electrode surface and the consumption of hydrogen peroxide, which led to mass transfer losses. The authors concluded that this hydrogen peroxide paper-based “green cell” had the potential of being integrated with self-sustainable, point-of-care diagnostic devices as a power source for (bio)sensing applications.

Yan et al. [81] demonstrated a simple design of a paper-based µ-fluidic HPFC. The CNT-supported rGO-PB cathode catalyst and Ag NWs were painted on the filter paper as the cathode and anode, respectively. The electrodes were contacted by means of conductive carbon paper, achieving an MPD of 0.88 mW cm^−2^ in 0.067 M HCl with 0.3 M H_2_O_2_ (Figure 6b). The cathode catalyst and Ag NWs were deposited as mixtures with Nafion. A continuous flow was maintained by a capillary action within the filter paper.

Zhou et al. [83] designed a flexible wire-shaped fuel cell (_W_FC) with a cathode comprising an Fe^(II)^_3_[Co^(III)^(CN)_6_]_2_ catalyst-loaded CNT yarn and an anode made of Ni NP-loaded CNT yarns placed in a silicone tubing with an acidic electrolyte (Figure 6c). The _W_FC achieved a power density of 6.28 mW cm^−2^, which was superior to that reported for the biofuel cells. The authors proposed using similar cells woven into textiles to power wearable electronics (Figure 6d). In 2024, the same group proposed a flexible coaxial HPFC in a silicon tubing with an acidic electrolyte (0.1 M HNO_3_, 0.15 M NaCl, and 0.1 M H_2_O_2_) [86] (Figure 6h,i). The authors studied carbon yarns composed of CNTs loaded with a C_60_-anchored Fe^(II)^_3_[Co^(III)^(CN)_6_]_2_ cathode catalyst in combination with an Ni or Ag wire as an anode. The cell with an Ni anode showed a higher OCV value (about 0.9 V), while the OCV of the cell with an Ag anode was slightly lower (about 0.8 V) but essentially more stable. The authors hypothesized that the Ni might have experienced a certain degree of corrosion, which became more pronounced over time. A high MPD value of 15.01 mW cm^−2^ was achieved by the 1.5 cm-long fiber HPFC.

A pumpless microfluidic HPFC was prepared on a cellulose paper using a “gel-aided” two-electrolyte configuration (Figure 6e) [87]. The ion-conductive hydrogel was sandwiched between a catholyte acidic flow (2 M HCl) and an anolyte alkaline (2 M KOH) flow. Using a high concentration of H_2_O_2_ (2 M), this design allowed the authors to reach an OCP value of 1 V and an MPD of 8.9 mW cm^−2^. The authors studied the mechanism of the cathodic and anodic processes and compared the OCP and power densities of the proposed “gel-aided” fuel cell with the characteristics of other HPFCs (Figure 6f). However, this design has limited applicability due to the requirement of two separate flows.

Wang et al. [85] demonstrated a flexible “on-fiber” H_2_O_2_ µ-fluidic FC (Figure 6g), which comprised a cathode made of MWCNT-PB-modified carbon fiber threads (CFTs) taken from carbon cloth and an anode made of Ni NP-modified CFTs. Three flexible cells were connected in series to power a calculator, thus demonstrating the possibility of being used as a power supply for an electronic device.

B. Nguen et al. [88] compared the performance of membraneless HPFCs with a series of transition metal phthalocyanines or Fe_x_N as a cathode electrocatalysts, and an Ni mesh anode under acidic conditions. It was found that CoPc had the lowest potential value for the onset of H_2_O_2_ reduction and the HPFC with a CoPc cathode electrocatalyst had the worst characteristic. The cell with the FePc cathode catalyst had a high OCP value of 0.56 V and produced the highest power density that was sufficient for operating microdevices, thus demonstrating the potential of the HPFC for producing sustainable energy sources. The cell with FePc cathode catalyst achieved an MPD of 3.41 mW cm^−2^, which was higher than the value in a previously reported study by Y. Yamada [64]. This difference might be due to the higher acidity and higher concentration of hydrogen peroxide used in [88]. An improved performance of a GNP-FePc nanocomposite was found in another study [67].

As can be seen from Table 1, most studies to date on the use of biomimetic and nanozyme catalysts for HPFCs have been conducted with cathode catalysts based on cyano-bridged transition metal complexes. The one-compartment HPFCs with PB and PB analogues reached an OCP of up to 0.9 V, which is close to the OCP of the two-compartment HPFC with Pt and Pd catalysts. Meanwhile, recent miniaturized flexible fiber-based cell designs have achieved MPD values of about 15 mW, thus approaching the MPD values of the two-compartment cells with Pt or Pd catalysts of about 21 mW cm^−2^ to 48.7 mW cm^−2^. Very few studies had been conducted on HPFCs with metallophthalocyanine cathode catalysts. At a pH of 1 to 7.4, those HPFCs achieved an OCP of 0.5 V to 0.6 V and a highest MPD of 3.41 mW cm^−2^ at pH 1, depending on the conditions [64,67,88]. The lower stability and poorer kinetics of the biomimetic catalysts, compared to catalysts such as Pt and Pd, therefore still need to be improved upon to achieve high power densities with biomimetic cathode catalysts. It should be emphasized that the acidic medium, which was used in the HPFC based on the PB cathode catalysts, is particularly favorable for these catalysts, since PB has a lower stability at a neutral and an alkaline pH [68,70,89]. Ni was used as an anode in most studies. Several studies used Ag, Ag_2_O, and Ag/AgCl as anodes. A lower OCP but better stability was achieved with an Ag anode [86]. However, no detailed comparative investigation of the potential-determining reaction and mechanisms was reported. A number of details on the behavior of the Ni anode can be found in [56,62,67,90]. One-compartment membraneless HPFCs based on a PB cathode catalyst in combination with Al and Mg anodes were also studied, demonstrating better performance than Ni anodes [80,91]. However, in these FCs, Al and Mg performed the role of reductants and the anode reaction was the oxidation of Al and Mg, while H_2_O_2_ was only an oxidant, distinguishing these “H_2_O_2_ semi-fuel cells” [91] from HPFCs, where H_2_O_2_ was used both as an oxidant and a reductant. An et al. [28] reviewed the H_2_O_2_ FC with various reductants.

Lower values of OCP of up to 0.35 V and an MPD of up to 374 µW cm^−2^ were obtained for the H_2_O_2_ FC operating at a pH of 7.4 with biological catalytic components such as hemin-based cathode catalysts and Co metal-organic compounds (Co(II)Pc, Co(II)Chl; Vit B12) as anode catalysts [92,93,94,95,96,97] (Table 1). Hashimoto et al. [98] recently reported an HPFC based on a combination of cyanobridged complex Fe(II)_3_[Co(III)(CN)_6_]_2_ supported on CNTs as a cathode catalyst with a Co(II)Chl supported on CNTs as an anode. However, the performance of the cell was lower than that of an HPFC with a similar cathode catalyst material and Ni or Ag anodes, even at a pH of 1 [83,85,86]. For comparison, wearable microbial BFCs achieved an MPD of up to 50 µW cm^−2^ [99], while recent enzymatic BFCs based on modifications of carbon nanomaterials achieved an MPD of up to about 1.1 mW cm^−2^, with a maximum current density of up to 200 µA cm^−2^ [100]. Yu et al. reported a lactate BFC that was capable of a power density of 3.4 mW cm^−2^ in an untreated body fluid [101], while BFCs typically achieve an energy output in the µW cm^−2^ to several mW cm^−2^ range and an output voltage of around 0.5 V [12,102].

It should be emphasized that the activity of the employed biomimetic and nanozyme catalysts has essential influence on the performance of HPFC; thus, one challenge is to increase the activity of biomimetic and nanozyme materials. To this end, different approaches have been investigated. It was found that due to a higher surface-to-volume ratio, materials with a smaller size have higher activity than larger ones. Meanwhile, since the catalytic center (metal ions) determines the catalytic properties, the composition, the morphology, and the surface atom arrangement of the aforementioned catalysts play key roles; thus, proper designs in the atomic arrangement and shape of the materials can be employed to enhance the exposure of the catalytic center, improve thermodynamics, and bring higher activity. Moreover, the long-term stability of biomimetic and nanozyme catalysts needs to be enhanced by surface modifications [35,37,39].

### 3.3. Photocatalytic HPFC

**Figure 7:**
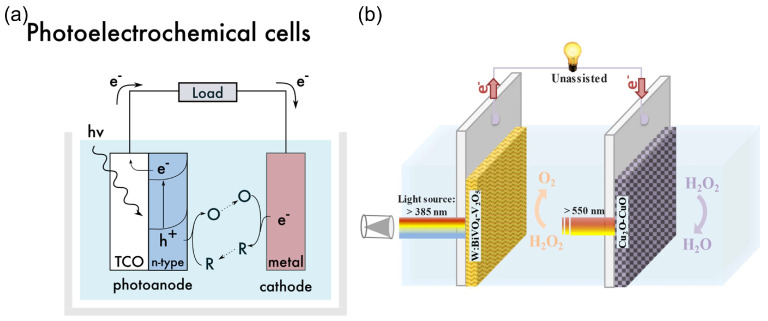
(**a**) Operating principle of a photoelectrochemical cell. TCO—transparent conductor. The design of the functional system requires particular energy levels of the semiconductor with respect to the redox potentials of the substances in an electrolyte. Reprinted from [12] with permission from Elsevier. (**b**) Schematic of a photocatalytic HPFC based on dual photoelectrodes [103]. Reproduced with permission from SNCSC.

A special type of HPFC is the photocatalytic HPFC (PC-HPFC), which generates electricity using sunlight or light sources [103,104]. The operating principle of the cell is illustrated in Figure 7a. In a PC-HPFC, photoactivation of the semiconductor active layers leads to the generation of charge carriers–electron–hole pairs. While the holes promote the oxidation of the reductant at the anode, the photogenerated electrons move through the external circuit to the cathode and participate in the reduction reaction of the oxidant, which results in output current. An example of the PC-HPFC proposed by Andrade et al. [103] is given in Figure 7b and Table 1. The cell was composed of a BiVO_4_ photoanode for hydrogen peroxide oxidation and a Cu_2_O photocathode for hydrogen peroxide reduction. It produced a short-circuit photocurrent density of 3.4 mA cm^−2^, an MPD of 0.26 mW cm^−2^, and an OCP of 0.48 V, with 0.2 M H_2_O_2_ in the acetate electrolyte. This PC-HPFC thus converted sunlight into electricity at the expense of carbon-free H_2_O_2_ acting as both a fuel and an oxidant.

**Table 1:** Hydrogen peroxide fuel cells and self-powered electrochemical sensors (application of HPFCs as H_2_O_2_ SPESs is highlighted in green. Photocatalytic HPFCs and H_2_O_2_ SPPhotoESs are highlighted in blue. The use of biological molecules as a cathode catalyst in an HPFC is highlighted in brown).

Anode	Cathode	pH, Electrolyte	H_2_O_2_, M Analyte, M	OCP, V	MPD, mW/cm^2^; (*MCD*, mA cm^−2^) ^a^	Configuration, One-/Two-Compartment	Sensor Application: DL; LR; S ^a^	Author, Year, Ref.
Pt	Pt	Anode: 1 M NaOH Cathode: 1.5 M H_2_SO_4_	0.75	0.7	23; (*80*)	2	–	Hasegawa et al., 2005 [46]
Au	Ag	1 M NaOH	0.3	0.095	–	1	–	Yamazaki et al., 2008 [62]
Au	Ag-Pb NP	1 M NaOH	0.3	0.15	0.075	1 ^b^	–	Yamada et al., 2010 [63]
Ni/C-paper	Pt/C	Anode: 6 M KOH Cathode: 1.5 M H_2_SO_4_	1 2	0.9	3.75	2	–	Sanli et al., 2011 [52]
Ni	Protonated FePc	pH 3, Acetate	0.3	0.5	10 µW cm^−2^	1	–	Yamada et al., 2011 [64]
Pt (H_2_O → O_2_)	PB NTs	pH 7, 0.1 M KCl Glucose	Up to 80 µM	–	About 30 µW cm^−2^	2, H_2_O_2_ in catholyte only	0.1 µM; up to 80 µM 0.048 A M cm^−2^; LR 1–25 mM	Wong et al., 2012 [65]
Ni Ag	PB	0.1 M HCl	0.5	0.6 0.53	1.55 0.8	1	–	Shaegh et al., 2012 [66]
d-Pd/CFC	d-Pd/CFC	Anode: 4 M KOH Cathode: 2 M H_2_SO_4_	1 2	0.9 0.9	14.3 (20 °C) 58.4 (60 °C)	2	–	Yang et al., 2012 [54]
d-Au-Pd/CFC	d-Au-Pd/CFC	Anode: 4 M KOH Cathode: 2 M H_2_SO_4_	1 2	0.9	20.7	2	–	Yang et al., 2013 [58]
Ni	Fe^(II)^_3_[Co^(III)^(CN)_6_]_2_ on CC	pH 3 (HClO_4_, 1 M NaCl) pH 1 (HClO_4_, 1 M NaCl)	0.3 0.3	0.68 0.78	0.45 1.2	1	–	Yamada et al., 2013 [74]
Ni/CFC	Pd/CFC	Anode: 4 M KOH Cathode: 2 M H_2_SO_4_	1 2	0.9 0.9	21.6 (20 °C) 53.8 (50 °C)	2	–	Yang et al., 2014 [55]
Ni	Fe(II)[Pt(CN)_6_](pyz) Fe^II^[Pd(CN)_6_](pyz)	pH 3, 1 M NaCl pH 1, 1 M NaCl pH 1, 1 M NaCl	0.3 0.3 0.3	0.8 0.7 0.78	0.09 4.2 4.2	1	–	Yamada et al., 2014 [75]
Mg (reductant)	PB	0.1 M HCl	0.5	2.3	7.5	1	–	Shaegh et al., 2014 [91]
	[Fe^II^(H_2_O)_2_]_3_[Co^(III)^(CN)_6_]_2_ therm. treated 60 °C	pH 1, 1 M NaCl, 0.12 M Sc^3+^	0.3	0.81	9.9	1	–	Yamada et al., 2015 [76]
Ni	Fe^III^(9-hydroxyphenalenone)_3_	0.1 M H_2_SO_4_	0.3	0.74	1.43	1	–	Pariyar et al., 2015 [77]
Ni NWA	Pd/CFC	Anode: 4 M KOH Cathode: 2 M H_2_SO_4_	0.9 2	0.9	48.7	2	–	Ye et al., 2015 [56]
Ni	Fe^(II)^_3_[Co^(III)^(CN)_6_]_2_ on CC	pH 1.3, seawater	0.048	0.78	1.6	1 ^c^	–	Mase et al., 2016 [105]
Ni Al (reductant)	PB	0.1 M HCl	0.5	0.32 0.61	0.38 0.81	1, µ-fluidic, paper/SU-8	–	Ehteshami et al., 2016 [80]
Ni@TiC NWA	Au-Pd/CFC	Anode: 4 M KOH Cathode: 2.0 M H_2_SO4	1 2	0.9	30.2	2	–	Wang et al., 2017 [57]
TiO_2_/Au NP/g-C_3_N_4_	PB	0.1 M PBS, pH 7.4 Ascorbic acid as an electron donor	–	–	–	1 ^d^	3.2 nM 0.005–200 µM	Wang et al., 2017 [106]
Not specified	ITO/Cu_2_O/CoP-NC	0.1 M PBS, pH 7.4 H_2_O is oxidized on anode	–	–	–	1 ^d^	0.1 µM 1–220 µM	Tian et al., 2017 [107]
FTO/mesoporous-TiO_2_	GCE	pH 3, 0.1 M NaClO_4_ (UV light)	0.1	0.72	(*0.24*)	1	–	Fujiwara et al., 2017 [104]
Ag/AgCl	PB/NiHCF	0.05 M PB pH 6, 0.1 M KCl H_2_O_2_ Glucose Lactate	0.2 µM–1 mM 5 µM–20 mM 0.5 µM–2 mM	–	–	1	A M cm^−2^ S_H2O2_ 0.65 S_glu_ 0.043 S_lact_ 0.18	Komkova et al., 2017 [108]
Ag NW	CNT-PB	1.5 M H_2_SO_4_	2	0.58	0.88	1, paper µ-fluidics	–	Yan et al., 2018 [81]
Ni	PEDOT	0.05 M HCl	0.1	0.56	0.31	1	–	Miglbauer et al., 2018 [78]
Bioanode from MFC	CoMn_2_O_4_ NPs/graphite	pH 7 300 Ohm	1–1000 mM	–	–	2 H_2_O_2_ in catholyte	40.2 µM 1–1000 mM 0.0132 A M^−1^	Liu et al., 2018 [109]
Bioanode from MFC	Graphite	pH 7 300 Ohm	1–2000 mM	–	–	2 H_2_O_2_ in catholyte	34.6 µM 1–2000 mM 0.011 A M^−1^	Liu et al., 2019 [110]
Pt	Au/PB	H_2_O H_2_O_2_ Glucose	Up to 0.2 mM 1–4 mM	0.11	1.2 µW cm^−2^ in 10 mM H_2_O_2_	1, IDE	0.02 µM up to 0.2 mM 0.035 A M cm^−2^ LR 1–4 mM	Ohnuki et al., 2019 [111]
Ni foam	rGO-PB on CC	0.67 M HCl	0.3	0.6	2.22	1, flexible µ-fluidics	–	Yang et al., 2019 [82]
CNT-Ni NPs	Biscrolled CNT-Fe^(II)^_3_[Co^(III)^(CN)_6_]_2_	0.1 M HClO_4_, 0.15 M NaCl	0.3	0.88	6.28	1, wire-shaped biscrolled yarns, _W_FC, flexible	–	Zhou et al., 2019 [83]
Ni	CoPc CuPc FePc Fe_x_N	0.1 M HCl	0.5	0.47 0.57 0.56 0.58	0.39 0.40 3.41 0.76	1	–	Nguyen et al., 2020 [88]
Ni	MWCNT-PB on CC	0.1 M HCl; 100 Ohm	0.5 0.25	0.66	5.5; (*34.1*) 2.7	1	Microfl. sensor 1.44 mM 5–50 mM 0.0375 A M^−1^	Liu et al., 2020 [84]
CNT/Vit B_12_	CNT/Hemin	0.01 M PBS, pH 7.4	0.1	0.233	53.8 µW cm^−2^	1	–	Ji et al., 2020 [92]
rGO/PAA/Co^II^Pc	[CNT/PEI]Hemin	0.01 M PBS, pH 7.4	0.1	0.260	72.1 µW cm^−2^	1	–	Ji et al., 2020 [93]
CNT/PEI/Co^II^Pc	CNT/PEI/Hemin (synthesis at 100 ° C)	0.01 M PBS, pH 7.4	0.1	0.340	129 µW cm^−2^	1, flow cell	–	Jeon et al., 2022 [94]
H-CoNC	CNT/PEI/Hemin	0.01 M PBS, pH 7.4	0.1	0.350	231.3 µW cm^−2^	1, flow cell	–	Ji et al., 2022 [95]
Ni NP/CFT	MWCNT-PB/CFT	0.3 M HCl	0.5	0.63	14.41	1, CFT, flexible	–	Wang et al., 2022 [85]
Ni	CNT-PB	Anode: 3 M KOH Cathode: 3 M HCl	2 M	1	10	2, paper, gel-aided dual-electrolyte	–	Luo et al., 2022 [87]
Porous g-C_3_N_4_/Ni	g-C_3_N_4_/FePc on CP	0.1 M HCl	0.3	0.626	0.248	1 ^e^	–	Li et al., 2022 [112]
BP/PEI/Co^II^Pc	BP/PEI/Hemin/	0.01 M PBS, pH 7.4	0.1	0.308	373 µW cm^−2^	1, flow cell	Porous separator, turbulent flow	Jeon et al., 2022 [97]
BP/Co^II^Pc	BP/Hemin/PEI/Hemin	0.01 M PBS, pH 7.4	0.1	0.171	90.7 µW cm^−2^	1, flow cell, flexible electrode materials	–	Jeon et al., 2023 [96]
W:BiVO_4_ -V_2_O_5_	Cu_2_O-CuO	1 M NaCH_3_COO	0.2	0.48	0.26; (*3.4*)	1	–	Andrade et al., 2023 [103]
CNT-Co^II^ chlorin	Fe^(II)^_3_[Co^(III)^(CN)_6_]_2_	pH 1, HClO_4_; 1 M NaCl	0.3	0.33	151 µW cm^−2^		–	Hashimoto et al., 2024 [98]
Ni	GNP-FePc	0.1 M PB pH 3 pH 7.4 pH 3, 10 kOhm	0.003	0.58	66 µW cm^−2^ at 20 kOhm	1	0.6 µM up to 3 mM 0.198 A M^−1^ cm^−1^ Blood Serum 0.2 µM up to 3 mM 0.197 A M^−1^ cm^−1^ 0.8 µM up to 1 mM 0.350 A M^−1^ cm^−1^	Zhang et al., 2024 [67]
Ni Ag	C_60_-ancored Fe^(II)^_3_[Co^(III)^(CN)6]_2_	0.1 M HNO_3_, 0.15 M NaCl	0.1	0.905 (Decline faster) 0.8 (Stable)	15.01; (35)	CNT fiber yarn, flexible	–	Zhou et al., 2024 [86]
Ni/PDI-Au NP	PDI/FePc	0.1 M HCl	0.2	0.7	1.07	1^e^	–	Li et al., 2024 [113]

^a^ MPD—maximum power density; *MCD*—maximum current density; DL—detection limit; LR—linear range; S—sensitivity; ^b^ coupled to solar H_2_O_2_ production to feed the fuel cell by the reduction of O_2_ with the electric power generated by a photovoltaic solar cell; ^c^ coupled to photocatalytic H_2_O_2_ production in seawater acidified to pH 1.3 to feed the fuel cell; ^d^ SPPhotoES converts solar energy to electricity, consuming H_2_O_2_ only as an oxidant; ^e^ coupled to solar H_2_O_2_ production; PB—phosphate buffer; d-Pd/CFC—dendritic Pd supported on carbon fiber cloth; CC—carbon cloth; pyz—pyrazine; NiHCF—nickel hexacyanoferrate stabilization layer; CNT—carbon nanotubes; rGO—reduced graphene oxide; NWA—nanowire arrays; GCE—glassy carbon electrode; CFT—carbon fiber thread; BP—bucky paper; CoNC—cobalt nitrogen-doped carbon; CoPc—cobalt phthalocyanine; CP—carbon paper; PAA—polyacrylic acid; H-CoNC—hollow cobalt nitrogen-doped carbon (Co–N bonds); PDI—perylene imide; CoP-NC—cobalt phosphide double-shelled nanocage.

### 3.4. Hydrogen Peroxide Fuel Cell with Sustainable H_2_O_2_

Following the principle of photosynthesis, converting solar energy into chemical energy using various technologies is a way of producing various energy resources. In this context, hydrogen peroxide can be produced from the earth-abundant environmental water and oxygen using sunlight as an energy supply. Different approaches have been used, such as photocatalytic, photoelectrochemical, and photovoltaic processes. The produced sustainable H_2_O_2_ can then be utilized in HPFCs and photocatalytic HPFCs. Several studies are discussed below to illustrate these approaches.

**Figure 8:**
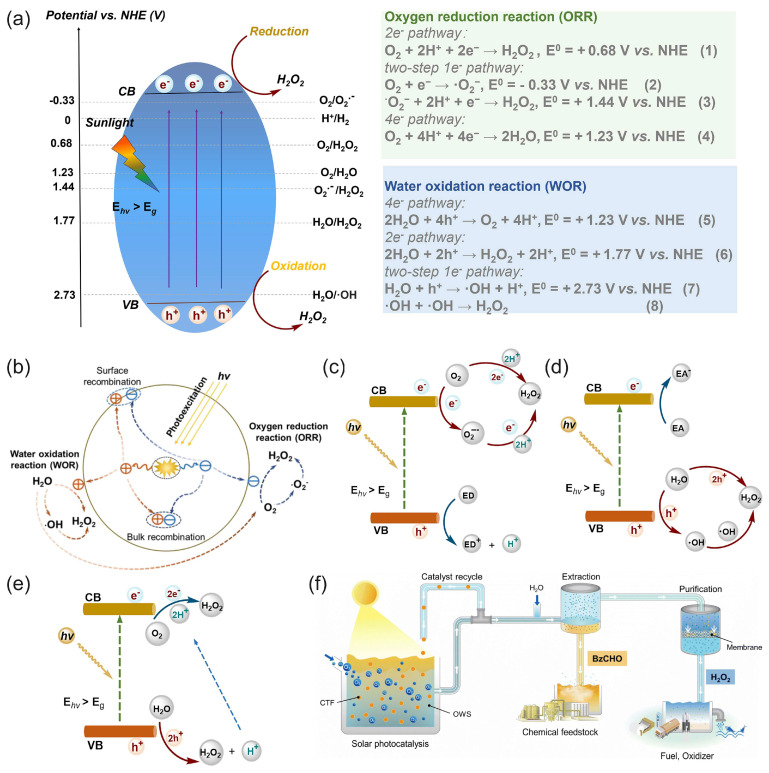
(**a**) Schematic of photocatalytic processes and an energy diagram of the oxygen reduction reaction (ORR) and water oxidation reaction (WOR) pathways for photocatalytic H_2_O_2_ production over semiconductor photocatalysts; potentials are given at pH = 0. CB—conduction band, VB—valence band, *E*_g_—bandgap, *E*_hν_—incident photon energy [50]. (**b**) Charge-transfer processes in the photocatalytic production of H_2_O_2_ from water and oxygen using a photocatalyst. Reproduced from [117] with permission from WILEY. Schemes of possible pathways for photocatalytic H_2_O_2_ production: (**c**) ORR pathway, (**d**) WOR pathway, and (**e**) dual-channel pathway. ED—electron donor, EA—electron acceptor. (**a**,**c**–**e**) Reproduced from [50] with permission from Elsevier. (**f**) Schematic of the photocatalytic solar production of H_2_O_2_ and benzaldehyde (BzCHO) with on-site applications. Reproduced from [115] and distributed under the terms of the Creative Commons license.

Solar energy can be directly used for the photocatalytic production (“photosynthesis”) of hydrogen peroxide through photocatalytic two-electron reactions of oxygen reduction or water oxidation with photocatalysts [48,50,114,115,116], as illustrated in Figure 8.

Zhu et al. [50] reviewed covalent organic frameworks as catalysts for photocatalytic production of hydrogen peroxide. Chen et al. [116] presented and discussed the design strategy of photocatalytic systems as well as the challenges and prospects related to photocatalytic production and applications of hydrogen peroxide. Moon et al. [115] recently proposed a new reaction design for solar H_2_O_2_ production using aryl alcohol as a photooxidation substrate and a covalent triazine framework as a metal-free polymeric photocatalyst with the quantitative generation of H_2_O_2_ by O_2_ reduction (Figure 8f). The photocatalytic system achieved an unprecedented H_2_O_2_ production rate of 46.9 mmol h^−1^ g^−1^ with a high solar-to-chemical conversion efficiency of 1.1% under simulated sunlight. The obtained H_2_O_2_ was further used for large-scale water purification as a proof of concept.

**Figure 9:**
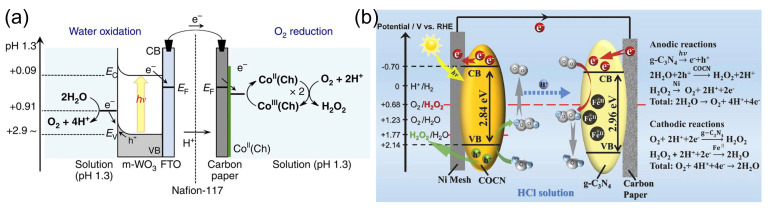
(**a**) Schematic of the photocatalytic production of H_2_O_2_ via water oxidation on the m-WO_3_/FTO photoanode and O_2_ reduction on the CoII(Ch)/CP cathode in acidified water or seawater. Reproduced from [105] and distributed under the terms of the Creative Commons CC BY license. (**b**) The mechanism of the photoelectrochemical cell with a g-C_3_N_4_/FePc cathode and an Ni mesh coated with porous g-C_3_N_4_ as the photoanode. Reproduced from [112] with permission from Elsevier.

Mase et al. [105] reported on the solar production of H_2_O_2_ in seawater followed by the utilization of the produced hydrogen peroxide in a fuel cell. However, two different cells were used in this work. Hydrogen peroxide was first photoelectrochemically produced from water and oxygen in a cell composed of an m-WO_3_/FTO photoanode and a cobalt–chlorin complex Co^II^(Ch)/glassy carbon cathode in acidified to pH 1.3 seawater under simulated 1 sun irradiation (Figure 9a). The chemical energy of hydrogen peroxide produced from the oxygen reduction reaction (ORR) in a cathode compartment was then converted to electrical energy using a one-compartment HPFC composed of a nickel mesh anode and an Fe^II^_3_[Co^III^(CN)_6_]_2_-modified carbon cloth cathode in acidified seawater with 48 mM of H_2_O_2_. The fuel cell achieved an OCP of 0.78V and an MPD of 1.6 mW cm^−2^. The solar-to-electricity conversion efficiency of the total system was about 0.28%, which was low compared to the conventional solar-to-electricity conversion of photovoltaic cells, which can reach a much higher efficiency of about 21% to 40%. Nevertheless, the authors concluded that the production of chemical energy from solar energy and its subsequent conversion to electrical energy based on H_2_O_2_ could provide a practical solution to an energy-sustainable society using seawater.

Li et al. [112] proposed a photoelectrochemical cell composed of a g-C_3_N_4_/FePc cathode and an Ni mesh coated with a porous g-C_3_N_4_ bearing structural defects of oxygen atoms and a COCN group as the photoanode (Figure 9b). When the electrodes were disconnected, the concentration of H_2_O_2_ photocatalytically produced by porous g-C_3_N_4_ reached 0.036 M in 0.1 M HCl under 1 sun irradiation for 3 h. The cell can store the hydrogen peroxide produced and then work as an HPFC using the obtained hydrogen peroxide to produce electricity by connecting the electrodes in the absence of light. This HPFC achieved an MPD of 0.248 mW cm^−2^ at 0.3 M H_2_O_2_ in 0.1 M HCl. The same group [113] later designed a photoelectrochemical system comprising perylene imide nanobelts (PDIs) decorated with AuNPs as the photoanode in combination with a PDI/FePc cathode. The incorporation of AuNPs resulted in the enhancement of photocatalytic H_2_O_2_ production through localized surface plasmon resonance effects.

In another approach, solar energy was used to generate electricity in a conventional photovoltaic cell. The photovoltaic electricity was used to perform the two-electron reduction of oxygen to hydrogen peroxide, and the hydrogen peroxide produced could then be used itself as both a reductant and an oxidant to generate electricity in an HPFC [49,63]. To illustrate this approach, Yamada et al. [63] reported a combination of H_2_O_2_ production through the electrocatalytic reduction of O_2_ in air using electrical power generated by a conventional Si photovoltaic solar cell and cobalt porphyrins as catalysts for O_2_ reduction, with subsequent power generation by a separate HPFC using an Ni anode and an Ag-Pb NP cathode catalyst. In this way, the produced H_2_O_2_ acted as a sustainable energy carrier, and the stored green energy could be released by a separate HPFC.

The mechanisms of water oxidation and oxygen reduction to produce hydrogen peroxide using photo-/electrocatalytic approaches, as well as recent advances in the design and synthesis of photo-/electrocatalysts and challenges for engineering photo-/electrocatalysts for H_2_O_2_ production, were reviewed in [118]. HPFC coupled to solar-driven photocatalytic and photovoltaic H_2_O_2_ production undoubtedly has potential as a sustainable energy source and as an alternative to other environmentally friendly carbon-free green fuels.

## 4. H_2_O_2_ SPES or H_2_O_2_ SPPhotoES Based on HPFCs

### 4.1. H_2_O_2_ SPES

Just as the research and development of enzymatic BFCs—following their invention by Yahiro et al. [119]—led to the realization of enzymatic SPBioESs, research and development in the field of HPFCs also led to attempts to use these configurations for H_2_O_2_ SPESs (Table 1), thus integrating research in the fields of fuel cells and electrochemical sensors [111]. Alongside the first applications of metal complexes as catalysts for the cathode of the HPFC [64,65,66], Wong et al. [65] used PB as a peroxidase mimetic material to demonstrate an H_2_O_2_ SPES and glucose SPBioES based on the H_2_O_2_ SPES (Table 1). However, in this sensor, an H_2_O_2_ analyte was not used as both an oxidant and reductant: instead, it was only added to the “sensing compartment” as an oxidant. The anode reaction delivering electrons to the circuit was supposed to be the oxidation of water on the Pt electrode. According to the proposed mechanism, the sensor thus had a two-compartment “galvanic cell” arrangement, with H_2_O_2_ acting only as an oxidant. This work also demonstrated the application of the two-compartment H_2_O_2_ SPES for the detection of mM concentrations of glucose, when coupled to the GOx-catalyzed oxidation of glucose in the catholyte solution.

Karyakin et al. [108] compared the performance of the PB/NiHCF-modified electrode as a sensor in a conventional three-electrode setup and in a “power generation” regime as a galvanic mode with the short-circuiting of the working and reference Ag/AgCl electrodes through the amperometer. The PB/NiHCF-modified electrode was prepared by layer-by-layer deposition of the Prussian blue and the stabilizing Ni hexacyanoferrate layers. In contrast to the high potential difference of cathodes and anodes in self-powered sensors, the authors proposed the system with the lowest potential difference. However, details about the anode part of the sensor and the mechanism were not discussed. Along with H_2_O_2_ sensing, glucose and lactate biosensing was demonstrated by immobilizing glucose oxidase and lactate oxidase on the surface of PB/NiHCF transducers, respectively. It was found that the sensitivities in a power generation mode were slightly higher and that the noise was an order of magnitude lower than in a conventional three-electrode setup.

Liu et al. [109,110] demonstrated the H_2_O_2_-sensing properties of a two-compartment FC using an anode from a microbial fuel cell (Table 1). H_2_O_2_ was introduced to the catholyte only and used as an oxidant. The sensors demonstrated detection limits of 40.2 µM and 34.6 µM, with sensitivities of 13.2 µA mM^−1^ and 11.0 µA mM^−1^, respectively. However, it should be emphasized that in the first H_2_O_2_ SPESs, hydrogen peroxide played the role of an oxidant, while the reducing substances on the anodes were different [65,108,109,110]. Thus, this kind of H_2_O_2_ SPES may require two-compartment configuration [65,109,110].

As discussed before, for H_2_O_2_ SPESs, one-compartment design is more attractive due to the simplicity of the devices and better compatibility for samples. To this end, different approaches have been proposed.

**Figure 10:**
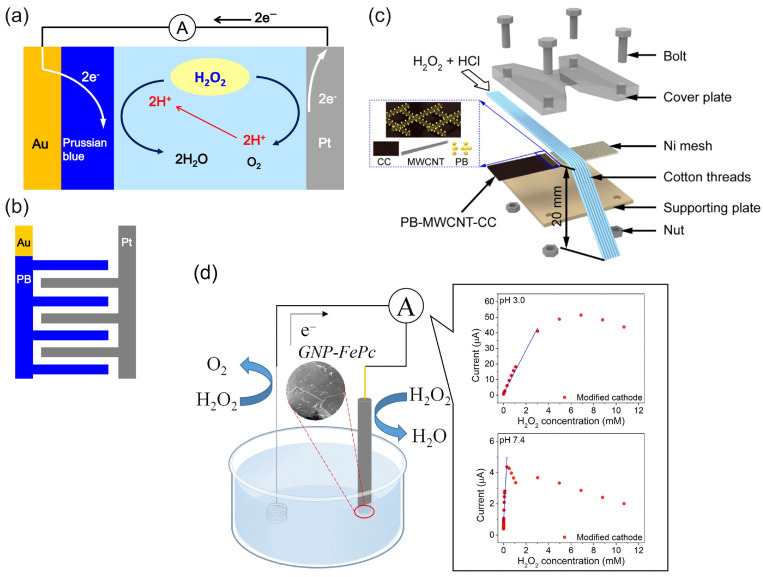
Examples of the H_2_O_2_ SPES with biomimetic and nanozyme catalysts: (**a**) schematic of an H_2_O_2_ SPES based on an array of (**b**) Au/PB–Pt IDE. (**a**,**b**) Used with the permission of IOP Publishing, Ltd., from [111]; permission conveyed through Copyright Clearance Center Inc. (**c**) Schematic of a single-stream microfluidic HPFC applied as a H_2_O_2_ SPES. Reproduced with permission from [84]. Copyright 2020 American Chemical Society. (**d**) Illustration of the H_2_O_2_ SPES based on an FC configuration with the biomimetic GNP–FePc cathode catalyst. Reproduced from [67] and distributed under the terms of the Creative Commons CC BY license.

Ohnuki et al. [111] reported modified interdigitated electrodes (IDEs) as a H_2_O_2_ SPES and its application as a glucose biosensor (Figure 10a,b). The IDE configuration allowed for a low detection limit (0.02 µM) of H_2_O_2_ to be achieved, with a linear concentration range of 0 to 0.2 M and a sensitivity of 0.0352 A M^−1^ cm^−2^. Glucose oxidase (GOx) was then immobilized on the Au/PB−Pt IDE by cross-linking with glutaraldehyde, and the enzymatic SPBioES demonstrated a dependence of the current density on the concentration of glucose due to the dependence of the H_2_O_2_ SPES signal on the concentration of H_2_O_2_ produced in the enzyme-catalyzed oxidation of glucose, in accordance with Equation (8):

(8) $$L\text{-}\mathrm{glucose}\;+\;O_{2}\;\frac{GOx}{\to }\;\mathrm{glucono}\text{-}\delta \text{-}\mathrm{lactone}\;+\;H_{2}O_{2}$$

Liu et al. [84] used a combination of the MWCNT-PB cathode catalyst and the Ni anode to demonstrate a microfluidic fuel cell and an H_2_O_2_ SPES working in a strongly acidic medium of 0.1 M HCl to achieve an OCP of 0.6 V (Figure 10c). The sensor had a linear range in the mM concentrations of H_2_O_2_ of 5 mM to 50 mM, with a detection limit of 1.44 mM, and a sensitivity of 0.0375 A M^−1^.

Zhang et al. [67] studied the mechanism and properties of the H_2_O_2_ SPES based on an FC configuration with biomimetic FePc and graphene nanoplatelets–FePc (GNP–FePc) cathode catalyst materials (Figure 10d). The authors performed a structural and electrochemical characterization of the modified cathodes as well as the Ni anode at pH 3 and pH 7.4. The best analytical characteristics of SPESs were achieved with the GNP–FePc cathode catalyst, with a larger working concentration range and significantly higher sensitivity towards H_2_O_2_ due to the improved electrochemical activity and reversibility of the Fe(III/II)Pc redox reaction on the GNP-modulated interface. More importantly, the practical applicability of the H_2_O_2_ SPES developed for the analysis of complex matrices was demonstrated by the good detection function of the concentration of hydrogen peroxide in a diluted (1:2 *v*/*v*) human serum. The H_2_O_2_ SPES demonstrated a better performance at pH 3 compared to pH 7.4, which was explained by the different protonation conditions and redox properties of iron phthalocyanine and the lower oxidizing properties of H_2_O_2_ at pH 7.4. At pH 3, the sensor had a sensitivity of 0.197 A M^−1^ cm^−2^ in a concentration range of 0.6 µM to 3 mM H_2_O_2_. It was unexpected that the introduction of an external resistor load resulted in an increase in the sensitivity to 0.350 A M^−1^ cm^−2^ due to the regulation of a current. The characteristics of the sensor under the control of a set of external variable load resistances indicated possible optimization routes. A decrease in the OCP of the cell was found with an increasing concentration of hydrogen peroxide. Although the detailed mechanism discussion depicted complex electrochemical properties and showed an interference effect from oxygen in a low H_2_O_2_ concentration range, this study highlights the simplicity in H_2_O_2_ SPES assembly and the improvement in the cathode interface, and, more importantly, presents the implementation and functional studies of a novel H_2_O_2_ SPES.

As discussed in the introduction, detection of the OCP dependence on the H_2_O_2_ concentration by the method of redox potentiometry may be advantageous for some applications. This detection mode was also tested for H_2_O_2_ SPESs [67] and for H_2_O_2_ detection in other applications [120,121].

As expected from thermodynamic considerations and discussed in the introduction, SPESs working in one compartment at mild conditions and low H_2_O_2_ concentrations, which is relevant for the applications of the sensor, do not typically achieve OCP and MPD values as high as those for HPFCs aimed purely at FC applications. Therefore, to overcome the ohmic resistance and polarization, it is essential to enhance the kinetics of the H_2_O_2_ redox reactions on both the cathode and the anode. Thus, even higher catalytic activities of the catalysts are required in H_2_O_2_ SPESs compared to in HPFCs for FC-only applications.

Table 2 shows some examples of conventional electrochemical sensors based on enzymes, nanozymes, and biomimetic materials operating in a three-electrode setup powered by potentiostat. Compared to the voltammetric and amperometric sensors powered by potentiostat, some of the SPESs reported to date (Table 1) already achieved comparable detection limits, working concentration ranges, and sensitivities. However, for electrochemical sensors powered by potentiostat, these parameters were achieved at the expense of energy provided in the form of a working potential applied to the working electrode, while operation of the SPESs is due to the spontaneous thermodynamically favorable reactions.

To further achieve higher sensitivity of SPESs, it is necessary to develop efficient catalytic materials with high rate constants of catalytic reaction and electron transfer with the electrode, as well as to increase the amount of immobilized electroactive catalyst molecules by using 3D nanomaterials to obtain a large active surface and exposure of catalyst sites. Some strategies were discussed in Section 3.2.

Unfortunately, the long-term stability of the HPFCs and SPESs is not profoundly discussed. At present, biomimetic and nanozyme catalysts such as phthalocyanine- and Prussian-blue-based materials are widely studied in HPFCs and SPESs as cathode materials, and an Ni anode is often employed. For one-compartment HPFCs and SPESs, the long-term stability is determined by the stability of the anode and cathode, and the main factor is the stability of the cathode materials. Due to the complicated mechanism and changes in the electrochemical processes of Prussian blue and phthalocyanine materials, the long-term stability of HPFCs and SPESs is limited. In particular, porphyrins and phthalocyanines may suffer from oxidative degradation, especially at high concentrations of hydrogen peroxide [67,73], while the operation stability of Prussian blue is limited, especially in neutral and alkaline conditions due to hydrolysis [68,70]. Thus, for practical application of HPFCs and SPESs, studies on increasing the stability of the materials are required, and the stability can be enhanced by surface modifications or composite design. For example, deposition of nickel hexacyanoferrate on the surface of Prussian blue was reported to improve the operation stability of the electrochemical sensors [70]. Nevertheless, Prussian blue and phthalocyanine materials are usually stable in the air at room temperature; thus, for sensor application, these materials can be employed for disposable sensors because the cost of Prussian blue and phthalocyanine materials is not high.

### 4.2. H_2_O_2_ Self-Powered Photoelectrochemical Sensors

H_2_O_2_ self-powered photoelectrochemical sensors (SPPhotoESs) use energy from sunlight or a light source to carry out photocatalytic electrochemical reactions in a cell typically comprising a semiconductor photoelectrode. The operational principle of SPPhotoESs is similar to the phototocatalytic HPFC discussed in Section 3.3, while an analyte or signal-forming substance participates in the redox reactions as a reductant or oxidant or influences the signal-forming redox process.

**Figure 11:**
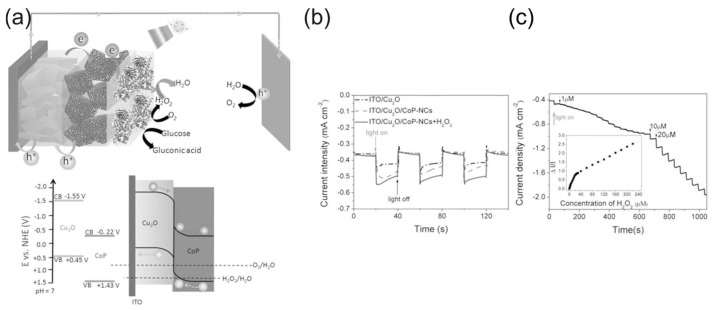
(**a**) The sensing mechanism of the SPPhotoES and the energy diagram of the photocathode; (**b**) photoresponse of ITO/Cu_2_O and ITO/Cu_2_O/CoP-NCs photocathodes in 0.1 M PBS (pH = 7.4) with and without 10 × 10^−6^ M H_2_O_2_; (**c**) photocurrent response of an ITO/Cu_2_O/CoP–NC photocathode to the addition of H_2_O_2_, with a bias potential of 0 V (against Ag/AgCl). Reproduced from [107] with permission from WILEY.

The operational principle of SPPhotoESs can be exemplified by the self-powered photoelectrochemical H_2_O_2_-sensing platform in Figure 11 [107]. Tian et al. [107] used broadband light-absorbing cobalt phosphide double-shelled nanocages (CoP–NCs) as a photoactive material for the construction of the photocathode for SPPhotoESs, detecting H_2_O_2_ using its reduction reaction and thus avoiding interference from physiological reductants such as ascorbic acid. The sensing mechanism of the SPPhotoES is illustrated in Figure 11. The photogenerated holes were transported to the anode via the VBs of CoP–NCs and Cu_2_O, and water oxidation was used as the anode reaction. The SPPhotoES had two linear response ranges—(1–20) × 10^−6^ and (20–220) × 10^−6^ M H_2_O_2_—with a detection limit of 0.1 µM. The SPPhotoES was further modified with glucose oxidase and used for the photoelectrochemical detection of glucose based on the quantification of the hydrogen peroxide produced during the oxidation of glucose by glucose oxidase.

## 5. Sensors Based on HPFCs or H_2_O_2_ SPESs

### 5.1. SPBioESs and Single-Enzyme SPBioESs Based on the Formation and Detection of H_2_O_2_

Hydrogen peroxide is a product of various reactions catalyzed by oxidoreductase enzymes in many important metabolic pathways, such as lactate oxidase, glucose oxidase, L-glutamate oxidase, and urate oxidase, according to reaction schemes (8) or (9):

(9) $$\mathrm{substrate}\;+{\;O}_{2}\;\frac{oxidase\;enzyme}{\to }\;\mathrm{oxidized}\;\mathrm{substrate}\;+{\;H}_{2}O_{2}$$

Consequently, there are many immuno- and enzymatic biosensors as well as enzymatic BFCs whose operation is based on the detection and use of hydrogen peroxide as a reaction product.

**Figure 12:**
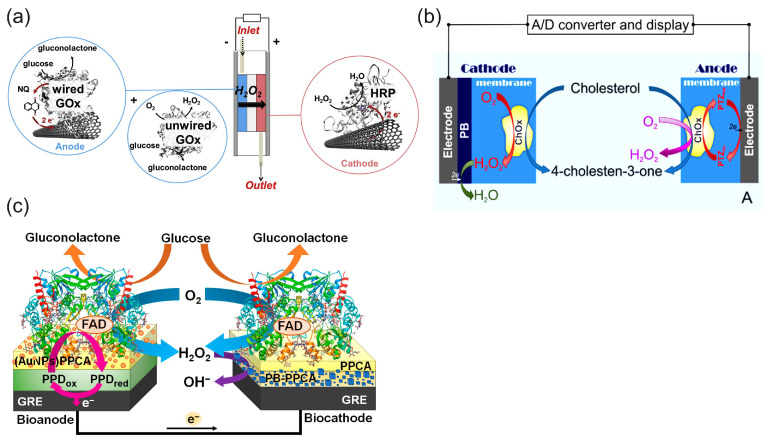
Schematic presentation of (**a**) the glucose/H_2_O_2_ flow-through BFC, where GOx is the glucose oxidase, NQ is the naphtoquinone electron transfer mediator, and HRP is the horseradish peroxidase. Reproduced from [30] with permission from Elsevier. (**b**) Single-enzyme SPES for the determination of cholesterol, where ChOx is the cholesterol oxidase, PTZ is a phenothiazine electron transfer mediator, and PB is the Prussian blue electrocatalyst. Reprinted with permission from [133]. Copyright 2014 American Chemical Society. (**c**) Single-enzyme SPES for the determination of glucose, where GRE is a graphite rod electrode, PPD is a poly(1,10-phenanthroline-5,6-dione) electron transfer mediator, PPCA is the poly(pyrrole-2-carboxylic acid) (PPCA) for the covalent binding of GOx on both electrodes, and PB on a cathode is the Prussian blue electrocatalyst. The sensor had a linear range of 0.15–124.00 mM, a sensitivity of 0.16 μA mM^−1^, and a detection limit of 0.07 mM. Reproduced from [134] and distributed under Creative Commons CC BY license.

Figure 12a illustrates the principle of a biofuel cell with the H_2_O_2_ reduction reaction on a biocathode in a bi-enzymatic system to replace oxygen as an oxidant and the oxygen reduction reaction on the cathode [30]. This design could help to solve the problem of oxygen cathodes in enzymatic BFCs in terms of the low concentration of oxygen and the possible deactivation of the oxidase enzymes of oxygen cathodes (LacOx, BOx) by the H_2_O_2_ produced in the anode reaction. In this BFC, wired GOx on the anode (a compressed mixture of MWCNTs, naphthoquinone as an electron-transfer mediator, and GOx) catalyzed the oxidation of glucose using mediated electrocatalysis and the electrons were transferred to the electrode by a naphthoquinone. At the same time, unwired GOx catalyzed the oxidation of glucose and reduced the natural electron acceptor, oxygen, to H_2_O_2_, according to Equation (8). The hydrogen peroxide produced was then transferred to the cathode by the constant directed flow produced—for example, by a peristaltic pump—and reduced to water by horseradish peroxidase at the cathode using the electrons delivered from the anode. It is worth noting that in the functioning mechanism of these BFCs, the hydrogen peroxide produced in the enzymatic reaction only played the role of an oxidant, while the reductant was an enzyme substrate. Oxygen was also necessary as a second oxidant and natural electron acceptor in the enzyme-catalyzed oxidation of the substrate. The design of BFCs based on the reduction of hydrogen peroxide has been expanded to other enzyme BFCs [17,79,102]. However, they require enzyme substrates as a fuel and oxygen as an additional oxidizer.

Due to the existing shortcomings of enzyme electrodes discussed in the introduction, the enzyme mimetic materials and nanozymes have tended to replace enzymes in BFC and SPBioES systems. This trend has been particularly successful in replacing HRP with cyano-bridged and N4 transition metal complexes, which offer an alternative to HRP for detecting the H_2_O_2_ reduction reaction and have led to the development of “single-enzyme” SPBioESs based on the reduction of hydrogen peroxide as a cathode process for the determination of analytes such as cholesterol and glucose [9,14,135]. Figure 12b illustrates the principle of a single-enzyme cholesterol SPBioES with PB as the peroxidase mimetic material for the H_2_O_2_ reduction reaction [133]. As both the anode and the cathode are modified with cholesterol oxidase, the use of only one enzyme in this design is particularly interesting. At the anode, cholesterol oxidase oxidizes cholesterol using mediated electron transfer. The electrons are transferred to the cathode via an external circuit to reduce Prussian blue to Prussian white, which is an electrocatalyst for hydrogen peroxide reduction. Hydrogen peroxide is also produced at the cathode due to the oxidation of cholesterol catalyzed by cholesterol oxidase using oxygen as a natural electron acceptor according to Equation (9). The sensor had a dynamic range for the detection of cholesterol of 0.15 mM to 4.1 mM, with a detection limit of 1.4 µM and a sensitivity of 26.0 mA M^−1^ cm^−2^ within the linear range.

Similar designs were also proposed for the single-enzyme glucose SPBioES [134,136] (Figure 12c). At the anode, the mediated electron transfer delivers electrons from the oxidized glucose to the cathode via an external circuit. At the cathode, a similar enzymatic reaction produces hydrogen peroxide according to Equation (9), which is electrocatalytically reduced by the PB/PW electrocatalyst.

Just like multiple-enzyme SPBioESs, “single-enzyme” SPBioESs use the substrate as a reductant and require oxygen as an additional oxidant. However, in comparison with multiple-enzyme sensors, the “single-enzyme” sensor may enable a simpler configuration and optimization of the BFC. It is worth noting that for electrochemical sensors, the generated current depends on the concentration of the analyte. Nevertheless, for enzyme SPBioESs, it needs be emphasized that the maximum reaction rate is also restricted by the enzyme itself. Thus, when the concentration of analyte reaches the saturation condition of the enzyme, the reaction rate and current are limited. Typically, for such sensors, the current depends on the concentration of the analyte up to a concentration of about K_m_ of the enzyme. As a result, these sensors are limited in application, with high concentration ranges of the analyte and high output power [14].

### 5.2. Wearable Systems Based on a Single-Enzyme SPBioES Using the Formation and Detection of H_2_O_2_ on Biomimetic Cathode Catalysts

**Figure 13:**
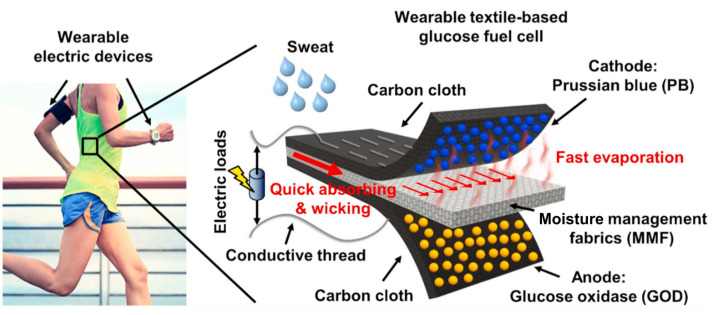
Illustration of the textile-based, single-enzyme BFC for the determination of glucose in the sportswear fabric. The electrons from the oxidation of glucose on the anode were transferred to the cathode via conductive threads. Hydrogen peroxide produced by the GOx-modified anode was transported to the cathode and reduced by Prussian white (the reduced form of PB) at the PB-modified cathode of the BFC. Reprinted from [137] with permission from Elsevier.

Single-enzyme SPBioESs with biomimetic cathode catalysts for the reduction of H_2_O_2_ have been used to develop flexible wearable systems in order to determine physiologically relevant metabolites. Wang et al. [137] developed a wearable and flexible single-enzyme glucose BFC integrated in the sportswear fabric with a GOx-modified anode and a PB-modified cathode for the H_2_O_2_ reduction reaction at the cathode (Figure 13). The OCV and maximum power of the multi-stack biofuel cell reached 1.08 V and 80.2 μW, respectively. A textile-based, six-stack BFC generated electrical power from human sweat and turned on a sports watch, thus demonstrating the ability to generate sustainable electrical power for wearable electronics using biofuel. This wearable BFC demonstrated a dependence of the current density on the concentration of glucose over a biologically relevant glucose concentration range of 0.1 mM to 100 mM, enabling its use as an H_2_O_2_ SPES.

**Figure 14:**
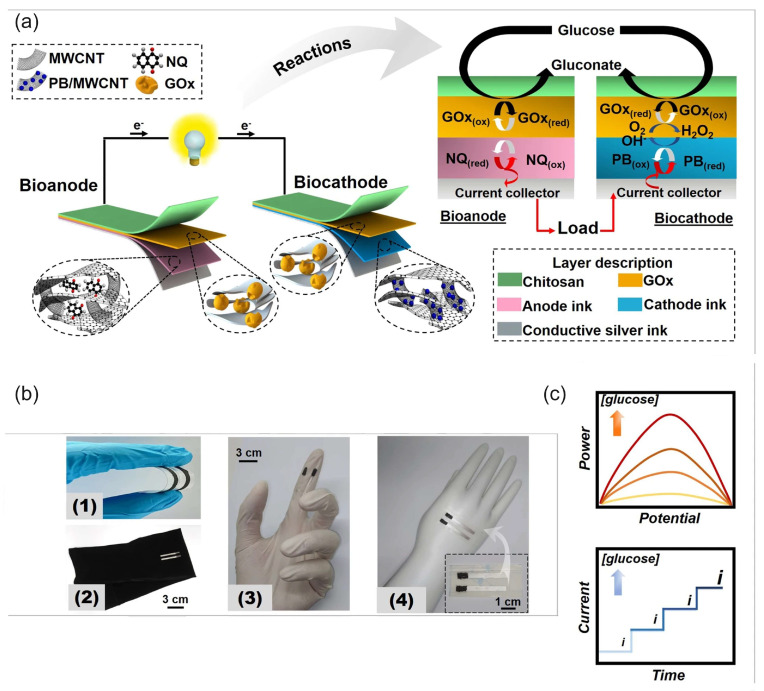
Schematic of a screen-printed, flexible single-enzyme system as a BFC and a glucose SPBioES: (**a**) the components of a screen-printed glucose BFC and redox reactions on the bioanode and the biocathode. (**b**) Images of a glucose single-enzyme BFC on (1) PET, (2) textile, (3) a glove, and (4) a stretchable epidermal tattoo. (**c**) Operation of a screen-printed glucose BFC in energy-harvesting (**top**) and self-powered sensing (**bottom**) modes. Reproduced from [136] and distributed under the terms of the Creative Commons CC BY license.

Veenuttranon et al. [136] engineered screen-printable nanocomposite inks for a flexible, single-enzyme-based energy-harvesting device and SPBioES powered by glucose on a bioanode and biocathode (Figure 14). The anode ink contained graphite, MWCNTs as conductive fillers, naphthoquinone (NQ) as a redox mediator, and polyurethane (PU) as a polymeric binder. The cathode ink consisted of graphite, a PB/MWCNT hybrid nanomaterial as an electrocatalyst, and PU. Both the bioanode and the biocathode were modified with GOx and consumed glucose, while the cathode also consumed oxygen, as illustrated in Figure 14a. This BFC achieved an OCV of 0.45 V and an MPD of 266 μW cm^−2^, as well as a current density of 1.3 mA cm^−2^ at a 20 mM concentration of glucose. As a SPEBioS, this cell could detect glucose concentrations of up to 10 mM, with a linear range of up to 1.5 mM of glucose and a sensitivity of 0.026 μA mM^−1^ cm^−2^. Common interfering substances—such as lactate, creatinine, ascorbic, and uric acids—had no effect on the SPEBioS. The H_2_O_2_ biomimetic cathode can thus substitute the O_2_ cathode in the wearable and single-enzyme SPBioES.

Since hydrogen peroxide is a product of biological processes, single-enzyme SPBioESs based on the reduction of hydrogen peroxide as a cathode process (HP-SPBioESs) represent an interesting area for biosensing applications, when using analytical markers of physiological importance for HP-SPEBioES operation. Due to an existing correlation of the concentrations of some of the biological analytes, such as glucose and lactate, in various biological matrices, such as tears, sweat, and interstitial fluid, with their blood or serum levels, these biological fluids have potential for biomedical diagnostics with HP-SPBioESs [138].

### 5.3. Self-Powered Electrochromic Sensors Based on Hydrogen Peroxide Detection—Optical Readout

Self-powered electrochromic sensors or self-powered displays use electrochromic materials to quantify the sensor output [139].

**Figure 15:**
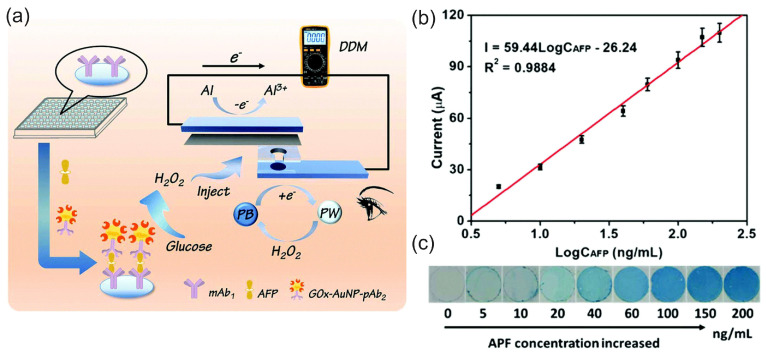
(**a**) Schematic representation of the self-powered aluminum/PB-based electrochromic display system for the detection of alpha-fetoprotein based on a sandwich-type enzyme immunoassay. The H_2_O_2_ produced in the GOx-catalyzed enzymatic oxidation of the added glucose results in a color change in the self-powered aluminum/PB-based electrochromic display. (**b**) Calibration curve of the self-powered electrochromic display sensing system towards AFP using current output measured with a multimeter. (**c**) Images showing the dependence of the color of the aluminum/PB-based, self-powered electrochromic display on the concentration of AFP. Used with the permission of the Royal Society of Chemistry, from [140]; permission conveyed through Copyright Clearance Center, Inc. (Danvers, MA, USA).

Figure 15 illustrates an enzyme immunoassay for the determination of alpha-fetoprotein (AFP) using an Al/PB-based, self-powered electrochromic display [140]. This type of cell has been described both in an FC configuration in Section 3.2 [80] and as a self-rechargeable battery [141]. The electrochromic sensor combined the Al/Al^3+^ anode half-cell reaction (Equation (10)) and the PB/PW cathode half-cell reaction (Equation (11)) in a galvanic cell with an overall reaction (12) [141].

Anode: Al → Al^3+^ + 3e^+^ (10)

Cathode: KFe^III^[Fe^II^(CN)_6_] (PB) + K^+^ + e^−^ → K_2_Fe^II^[Fe^II^(CN)_6_] (PW) (11)

3KFe^III^[Fe^II^(CN)_6_] (PB) + 3K^+^ + Al → 3K_2_Fe^II^[Fe^II^(CN)_6_] (PW) + Al^3+^ (12)

As a result of reaction (12), the cathode of the detection cell possessed nearly transparent PW on its surface. Then, the authors used a sandwich-type immunoreaction on a microtiter plate coated with monoclonal antibodies against AFP (mAb1) and an anti-AFP polyclonal antibody labeled with gold nanoparticles with the attached GOx (GOx-AuNP-pAb2) as the detection antibody. In the presence of the immunoreaction and the addition of the glucose, the attached GOx catalyzed the oxidation of glucose by oxygen, with the formation of hydrogen peroxide according to Equation (8). As the concentration of AFP analyte affected the reaction rate of glucose oxidation, the amount of produced hydrogen peroxide was therefore AFP concentration-related. Then, the introduction of the produced hydrogen peroxide into the detection cell resulted in the oxidation of the almost colorless PW back to PB, according to Equation (13), meaning that the battery could be recharged and reactions (10) and (11) could proceed again, and the current could be amplified.

2K_2_Fe^II^[Fe^II^(CN)_6_] (PW) + H_2_O_2_ → 2KFe^III^[Fe^II^(CN)_6_] (PB) + 2K^+^ + 2OH^−^ (13)

Meanwhile, the oxidation of the colorless PW to blue PB by the produced hydrogen peroxide resulted in a visual color change in the self-powered aluminum/PB-based electrochromic display system, which can be used for qualitative detection with the naked eye. In addition, H_2_O_2_ can amplify the current output, which can be quantified by a multimeter. Thus, this work demonstrates the principle of a simple and portable self-powered electrochromic display for point-of-care devices based on the formation and consumption of hydrogen peroxide.

Based on the reported studies, it can be concluded that since hydrogen peroxide is naturally present in biological systems, the SPES and SPBioES based on the H_2_O_2_ SPES have the potential to be used as wearable and implantable sensors and power supply devices to integrate with other components, including wireless communication units and interconnects, for health monitoring and multiparametric sensor and actuator networks [142].

## 6. Conclusions and Outlook

The article provides an overview of research and development in the field of self-powered electrochemical systems based on the use and detection of hydrogen peroxide according to the principle of galvanic cells or photoelectrochemical cells. Additional aspects of sustainable hydrogen peroxide fuel cells are discussed as well as SPPhotoESs, single-enzyme SPBioESs, the replacement of the oxygen cathode, and SPESs with an optical readout. A description of different cell designs is also given in addition to flexible fiber and textile cells, paper-based microfluidic approaches, and their corresponding cell performance. It is stressed that along with the cell design and electrolytes, cathode and anode catalysts are a key technology for the characteristics and performance of hydrogen peroxide fuel cells and the SPESs based on them.

Despite developments in the field of hydrogen peroxide SPESs, the choice and properties of the materials for the non-enzymatic cathode remain limited. As a result, hydrogen peroxide fuel cells and SPESs based on metal catalysts mainly involve a two-compartment design. Hydrogen peroxide fuel cells and SPESs based on nanozymes and biomimetic cathode catalysts are limited to the use of cyanometallates or phthalocyanine complexes. As a result, they exhibit better stability and properties at a low pH, but the insufficient oxygen selectivity may limit their use in biomedical research. In contrast to the extensive research on cathode materials, only a few studies have been conducted on anode materials and processes. However, as the anodic reaction is also a key factor to the electrochemical system, for insightful understanding of the electrochemical processes and to achieve better performance, it is necessary to pay more attention to the anode side.

Based on the current progress in hydrogen peroxide SPESs, it is anticipated that the detection of various biological and environmental markers and industrial analytes can be performed using modified electrodes and H_2_O_2_ produced in a signal-forming reaction with its quantification by hydrogen peroxide SPESs. Further research on catalyst development, flexible platforms, and designs, and the study of the mechanisms of hydrogen peroxide fuel cells, are expected to enable the elaboration of H_2_O_2_ SPESs and SPBioESs based on an H_2_O_2_ SPES, with better performance for a wide range of applications such as sustainable applications, smart textiles with power sources and sensor networks, personalized healthcare, and remote monitoring.

## Figures and Tables

**Table 2:** Electrochemical H_2_O_2_ sensors using a three-electrode setup powered by potentiostat.

Electrode	E, V	Linear Range, mM	Sensitivity, A/(M⋅cm^2^)	DL, µM	pH, Electrolyte	Application	Ref.
GCE/Fe_3_O_4_@rGO/Hemoglobin/Nafion	−0.28	0.0015–2.68	0.0181 A M^−1^	1.4	pH 7.0	Blood serum	[122]
SPCE/MWCNT, Au NP, PAni/catalase	−0.4	0.01–6.8	0.0588	2.34	pH 7.4	Milk	[123]
GCE/COF, HRP	−0.2	0.00953–0.007	–	0.00281	pH 7.0	–	[124]
GCE/PEDOT/Cu(II)O NP	−0.4	0.04–10	4.6 A M^−1^	8.5	0.1 M NaOH	Milk	[125]
Pd-Au NW	−0.05	0.001–1	18 µA M^−1^	0.3	pH 7.2	HL1 cells	[24]
GCE/PAni, CeO_2_ NP	>0.7	0.002–0.1	–	0.15	pH 5.7	Milk, water	[126]
GCE/MWCNT-Ti_3_C_2_T_x_-Pd NP	0	0.05–18	0.294	3.83	PBS	*Arabidopsis*	[127]
GCE/Fe_3_O_4_ NP @ZIF-8-MoS_2_, Au NS, Nafion	−0.55	0.005–15 15–120	0.0171 A M^−1^ 0.00417 A M^−1^	0.9	pH 7.4	H9C2 cells	[128]
GCE/MnO-Mn_3_O_4_ MP @rGO	−0.45	0.004–17	247.15	0.1	0.2 M NaOH	Tomato sauce	[129]
BDD/PB	0	10^−4^–1	0.14	–	pH 6, 0.1 M KCl	–	[130]
GCE/Mn-PB NS/Nafion	−0.05	0.002–10 0.003–0.7	0.12 0.14	2 3	pH 3 pH 7.4	–	[69]
GCE/rGO, PB NP/Nafion	0	0.0012–5 0.006–1	0.09 0.06	1.2 6	pH 3 pH 7	–	[89]
Au ND/rGO/MnTMPyP/Nafion	−0.45	3.4⋅10^−4^–0.015 0.015–0.08	0.384 0.07	0.3	pH 7.4	Plant leaves	[131]
GCE/COF based on porphyrin and Fe^2+^	−0.2	6.85⋅10^−6^–0.007	7.3	0.002	pH 7	–	[132]

PAni—polyaniline; COF—covalent–organic framework; BDD—boron-doped diamond; NS—nanostructures; Mn-PB—Mn-doped PB.

## Data Availability

Data were obtained and reproduced from copyright holders with permission or reproduced from materials distributed under the terms of the Creative Commons CC BY license. Permission were obtained through Copyright Clearance Center, Inc.
